# Rapid MALDI-MS/MS-Based Profiling of Lipid A Species from Gram-Negative Bacteria Utilizing Trapped Ion Mobility Spectrometry and mzmine

**DOI:** 10.1021/acs.analchem.4c05989

**Published:** 2025-04-01

**Authors:** Edward Rudt, Matti Froning, Steffen Heuckeroth, Lucas Ortmann, Julia Diemand, Linus Hörnschemeyer, Alexander Pleger, Max Vinzelberg, Robin Schmid, Tomáš Pluskal, Ulrich Dobrindt, Heiko Hayen, Ansgar Korf

**Affiliations:** † Institute of Inorganic and Analytical Chemistry, University of Münster, Corrensstraße 48, D-48149 Münster, Germany; ‡ mzio GmbH, Altenwall 26, D-28195 Bremen, Germany; § Institute of Organic Chemistry and Biochemistry of the Czech Academy of Sciences, Flemingovo náméstí 542/2, 160 00 Prague, Czech Republic; d Institute of Hygiene, University of Münster, Mendelstraße 7, D-48149 Münster, Germany

## Abstract

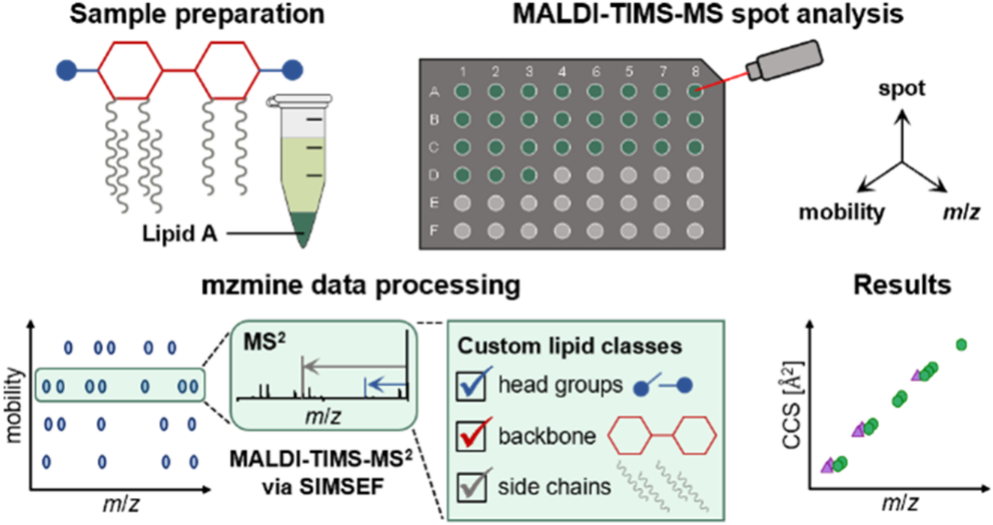

Lipid A, a crucial component of lipopolysaccharides (LPS), plays a pivotal role in the pathogenesis of Gram-negative bacteria. Lipid A patterns are recognized by mammals and can induce immunostimulatory effects. However, the outcome of the interaction is highly dependent on the chemical composition of individual lipid A species. The diversity of potential fatty acyl and polar headgroup combinations in this complex saccharolipid presents a significant analytical challenge. Current mass spectrometry (MS)-based lipid A methods are focused on either direct matrix-assisted laser desorption/ionization (MALDI)-MS screening or comprehensive structural elucidation by tandem mass spectrometry (MS/MS) hyphenated with separation techniques. In this study, we developed an alternative workflow for rapid lipid A profiling covering the entire analysis pipeline from sample preparation to data analysis. This workflow is based on microextraction and subsequent MALDI-MS/MS analysis of uropathogenic *Escherichia coli* utilizing trapped ion mobility spectrometry (TIMS), followed by mzmine data processing. The additional TIMS dimension served for enhanced sensitivity, selectivity, and structural elucidation through mobility-resolved fragmentation via parallel accumulation-serial fragmentation (PASEF) in parallel reaction monitoring (prm)-mode. Furthermore, mzmine enabled automated MS/MS acquisition by adapting the spatial ion mobility-scheduled exhaustive fragmentation (SIMSEF) strategy for MALDI spot analysis. It also facilitated robust lipid A annotation through a newly developed extension of the rule-based lipid annotation module, allowing for the custom generation of lipid classes, including specific fragmentation rules. In this study, the first publication of lipid A species’ collision cross section (CCS) values is reported, which will enhance high-confidence lipid A annotation in future studies.

Lipopolysaccharides (LPS) are integral components of the outer cell membrane of Gram-negative bacteria, such as *Escherichia coli*.^1,2^ The structure of LPS can be divided into three sections: the *O*-polysaccharide or *O*-antigen, which consists of repeating sugar units; the oligosaccharide core, which is subdivided into an inner and outer core; and the lipid anchor, called lipid A.^1,3^ LPS are recognized in mammals by interaction with the Toll-like receptor 4 complex and can have strong immunostimulatory effects, dependent on the LPS structure.^1,2^

The pathological effect of LPS as endotoxins is primarily caused by its anchor, lipid A.^3^ In this context, the composition of lipid A can exert a tremendous influence on bacterial endotoxicity.^3,4^ In general, lipid A is composed of an β-(1,6)-linked glucosamine backbone with varying polar head groups and fatty acid residues. The fatty acid residues are bound to different sugar backbone positions (2′, 3′, 2, and 3) as primary acyl chains, and may be further branched by secondary acyl chains.^1,3^ The polar head groups exhibit different levels of phosphorylation (1 and 4′ positions) that can undergo further modification, e.g., by phosphoethanolamine (PEtN) or aminoarabinose (AraN). The number, position, and length of bound fatty acids, as well as the number, position, and type of polar head groups, can differ between bacterial strains.^5,6^ Variation in lipid A composition has been observed in strains from different bacterial genera^6,7^ or even in strains within the same genus. This diversity results from the adaptation of lipid A to harmful environmental conditions to ensure cell survival.^6,8^

In the case of the model organism *E. coli*, the major lipid A species consists of six fatty acids including two secondary acyl chains and two phosphate groups.^9^ However, additional lipid A species of lower abundance were detected in *E. coli*, exhibiting variations in the degree of acylation, in the length of bound fatty acids, and in the phosphorylation level.^10−13^ This highlights the structural diversity of lipid A species, making lipid A characterization analytically challenging. Consequently, selective and sensitive methods are required for their structural elucidation.

Several mass spectrometry (MS)-based techniques have emerged as powerful tools for lipid A profiling. Direct analysis by matrix-assisted laser desorption/ionization (MALDI)-MS,^14,15^ or comprehensive analysis by combining a separation technique with electrospray ionization (ESI)- tandem mass spectrometry (MS/MS),^10,11^ are the most commonly used techniques to study lipid A composition. Although direct MALDI-MS offers rapid analysis,^16^ straightforward microextraction,^17^ and potential for bacterial screening,^14^ it faces challenges, such as lower sensitivity caused by ion suppression effects, compared to, e.g., high-performance liquid chromatography (HPLC)-MS, and the lack of automatized structural elucidation. In contrast, HPLC,^10,11^ capillary electrophoresis (CE),^18,19^ or supercritical fluid chromatography (SFC)^20^ hyphenated to MS/MS enables comprehensive analysis of the entire lipid A profile through its advantageous selectivity and sensitivity. However, this approach is more time-consuming and requires an extensive sample preparation with numerous extraction and purification steps.^21^

An alternative rapid separation technique is ion mobility spectrometry (IMS).^22,23^ In addition to the selective mobility separation based on size, charge, and shape,^24^ the analyte-specific collision cross section (CCS) provides valuable data that facilitates correct lipid annotation in IMS-MS.^25^ While IMS is widely used in lipidomics,^26^ lipid A research lacks such applications. Several advantages were shown for IMS in lipidomics, such as the use of high-confidence lipid annotation based on improved selectivity in combination with CCS databases,^27,28^ the resolution of isobaric and isomeric interferences,^29,30^ and the increased sensitivity for quantitation by noise-filtering.^31,32^ IMS offers particular advantages for methods that do not allow chromatographic hyphenation, such as imaging.^33,34^ In MALDI-MS imaging, the postionization and high resolution-IMS technique of trapped ion mobility spectrometry (TIMS) is widely used.^35,36^ Usage of TIMS adds an additional separation dimension capable of resolving distinct lipid isomers and isobars,^37^ as well as the ability to fragment these interferences in a mobility-resolved manner via parallel accumulation-serial fragmentation (PASEF).^38,39^ In recent years, parallel reaction monitoring (prm)-PASEF has been introduced as a targeted PASEF approach.^40^ Thereby, prm-PASEF has shown distinct benefits due to its reproducible and comprehensive scheduling, leading to high sensitivity in fragmentation.^41^ More recently, prm-PASEF was adapted to MALDI-MS/MS^42,43^ and combined with an automated acquisition strategy for data set-dependent fragmentation in imaging called spatial ion mobility-scheduled exhaustive fragmentation (SIMSEF). This automation facilitates precursor selection, enabling confident on-tissue lipid annotation of major lipids on species or molecular species level.^44^

Due to the many advantages of TIMS in lipidomics, and the high speed of MALDI-MS for bacterial screening purposes, we developed a workflow for lipid A profiling based on microextraction and subsequent MALDI-TIMS-MS/MS analysis via prm-PASEF. In addition, the SIMSEF acquisition strategy^44^ was adapted for automated, data set-dependent fragmentation in MALDI spot analysis. As proof of concept, uropathogenic *E. coli* strain CFT073^45^ was analyzed, which was prepared either shock-frozen or autoclaved. To confirm the results, the obtained CCS values were compared with those generated by LC-TIMS-MS/MS. All data sets were analyzed with mzmine^46^ for fast and robust lipid A annotation in both LC-TIMS-MS/MS and MALDI-TIMS-MS/MS data sets. For that purpose, the mzmine rule-based lipid annotation module was extended, allowing for the custom generation of lipid classes including specific fragmentation rules.

## Experimental Section

### Chemicals

Authentic standards of synthetic monophosphorylated lipid A (MPLA) and diphosphorylated lipid A (DPLA) from *E. coli* strain F583 as well as phosphate buffered saline (PBS), ammonium formate (NH_4_FA, ≥99.995%), trisodium citrate dihydrate (≥99.5%), *trans*-ferulic acid (FA, 99%), and 9-aminoacridine (9-AA, ≥99.5%) were purchased from Sigma-Aldrich (Steinheim, Germany). Formic acid was obtained from Th. Geyer (Renningen, Germany). LC-MS grade 2-propanol and methanol were purchased from VWR International (Darmstadt, Germany). HPLC grade chloroform was acquired from Merck (Darmstadt, Germany). Citric acid (≥99.5%) was obtained from Honeywell (Seelze, Germany). The Milli-Q eq 7000-system from Merck (Darmstadt, Germany) was used to obtain purified water.

### Cultivation

*E. coli* strain CFT073^45^ was cultivated at 37 °C in batch cultures in M9 minimal medium supplemented with 0.2% glucose for 16 h (three biological replicates). The bacterial cell pellets were subsequently either shock-frozen or autoclaved additionally and stored at −80 °C. The optical density (OD_600_) of the cultures at the time of harvest was determined and is listed in the Supporting Information (SI-1). Prior to further sample preparation, cell death of the bacteria was confirmed.

### Sample Preparation

Lipid A extraction for LC-MS/MS and MALDI-MS/MS was optimized based on modified protocols of Henderson et al.^21^ and Sorensen et al.,^17^ respectively. The samples were treated identically, dried, and weighed, followed by different protocols depending on the applied method. Details of MALDI-MS/MS microextraction and extensive LC-MS/MS sample preparation are provided in the Supporting Information (SI-2).

### Instrumental Setup

MS measurements were performed on a timsTOF fleX instrument (Bruker Daltonics GmbH & Co. KG, Bremen, Germany), including an Apollo II source for ESI measurements and a Nd:YAG smartbeam three-dimensional (3D) laser at 355 nm for MALDI. Measurements were conducted in both negative and positive ionization modes. For LC-TIMS-MS/MS, a Vanquish Flex UHPLC system (Thermo Scientific, Dreieich, Germany) was used, equipped with a Nucleodur Sphinx column (50 mm × 2.0 mm, 1.8 μm; Macherey-Nagel, Düren, Germany). Detailed information on the instrumental setup for LC-TIMS-MS/MS and MALDI-TIMS-MS/MS including SIMSEF acquisition is described in the Supporting Information (SI-3).

### Data Processing

For data evaluation, the open-source software mzmine 4.1 software^46^ (mzio GmbH, Bremen, Germany) was utilized. Individual batch files were created for MALDI-TIMS-MS/MS and LC-TIMS-MS/MS in both positive and negative ionization mode, which were subsequently aligned to validate the results. Additionally, data evaluation was confirmed in DataAnalysis 6.1 (Bruker Daltonics GmbH & Co. KG, Bremen, Germany). The batch files are provided as mzbatch files (doi:10.25345/C5707X11F) and in written form in the Supporting Information (SI-4) along with the custom lipid A classes.

## Results and Discussion

### MALDI-TIMS-MS Method Development

The aim of this study was to develop an efficient and straightforward MALDI-TIMS-MS/MS method for comprehensive lipid A screening in bacterial samples. For this purpose, the MALDI approach was optimized, and a tube-based microextraction for subsequent MALDI spot analysis was developed, as described thoroughly in the Supporting Information (SI-5).

### Additional TIMS Dimension in Lipid A Profiling

In addition to the development and optimization of the MALDI approach, the benefits of TIMS for comprehensive lipid A profiling were investigated. TIMS has been established as a reliable tool for high-confidence annotation in lipidomics.^47,48^ However, there is a lack of TIMS applications for lipid A profiling in the literature. For method development, *E. coli* CFT073 and lipid A standards were utilized.

In addition to the separation of isomeric compounds, TIMS can be utilized as a mobility filter. Thus, statistical noise can be reduced and interfering compounds may be distinguished based on the CCS, which may result in enhanced sensitivity.^31,32^ In the case of lipid A, the high CCS values are atypical for metabolites and require an adjustment of the pressure in the TIMS cartridge for acquisition. As a result of the high CCS value, the influence of mobility filtering on the sensitivity of MALDI-TIMS-MS analysis was investigated. In fact, TIMS serves to enhance the sensitivity for lipid A profiling by noise-filtering (Figure S5). Moreover, the high number of unknown and interfering compounds has been reduced considerably, especially in positive ionization mode. The mobility-filtering will also lead to a reduction in the number of false positives in software-based lipid annotation.

**Figure 1:**
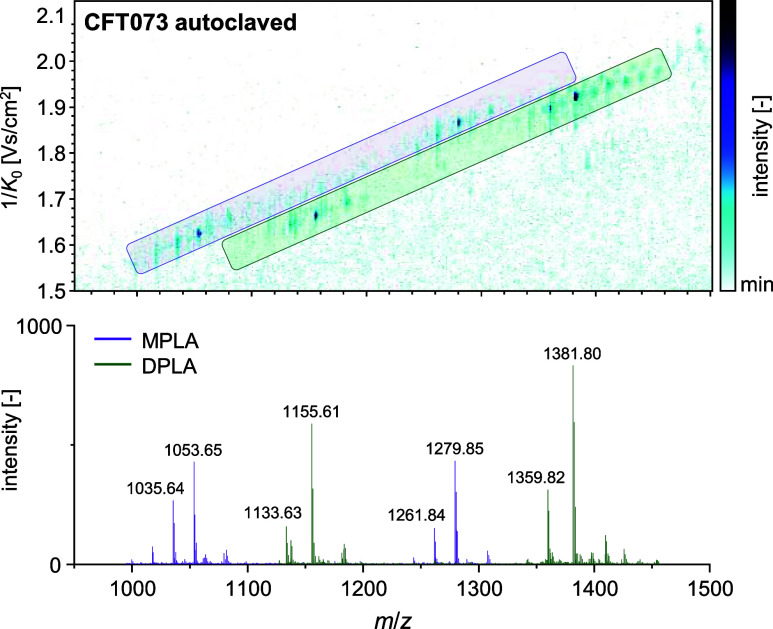
Heatmap of the detected ions in autoclaved *E. coli* CFT073 using MALDI-TIMS-MS in negative ionization mode. The trajectories of MPLA and DPLA are highlighted, and overlaid color-coded mass spectra of these regions are plotted below.

Further reduction of false positive annotations may be achieved by implementing CCS relationships depending on changes in the molecular structure of lipid A. Therefore, the obtained mobility values of the lipid A species in autoclaved *E. coli* CFT073 in negative ionization mode were subjected to a more detailed examination (Figure 1).

The mass/mobility plot revealed distinct differences in the trajectory of 3-acyl and 4-acyl lipid A based on the level of phosphorylation and independent of the adduct formation (i.e., [M – H]^−^ and [M + Na – 2H]^−^, such as *m*/*z* 1133.63 and *m*/*z* 1155.61, respectively). Thus, different trajectories were detected for MPLA and DPLA, which will facilitate the correct lipid annotation of these lipid A classes. Therefore, TIMS not only allows for a reduction of potentially interfering unknowns but also serves to enhance selectivity and increase sensitivity.

**Figure 2:**
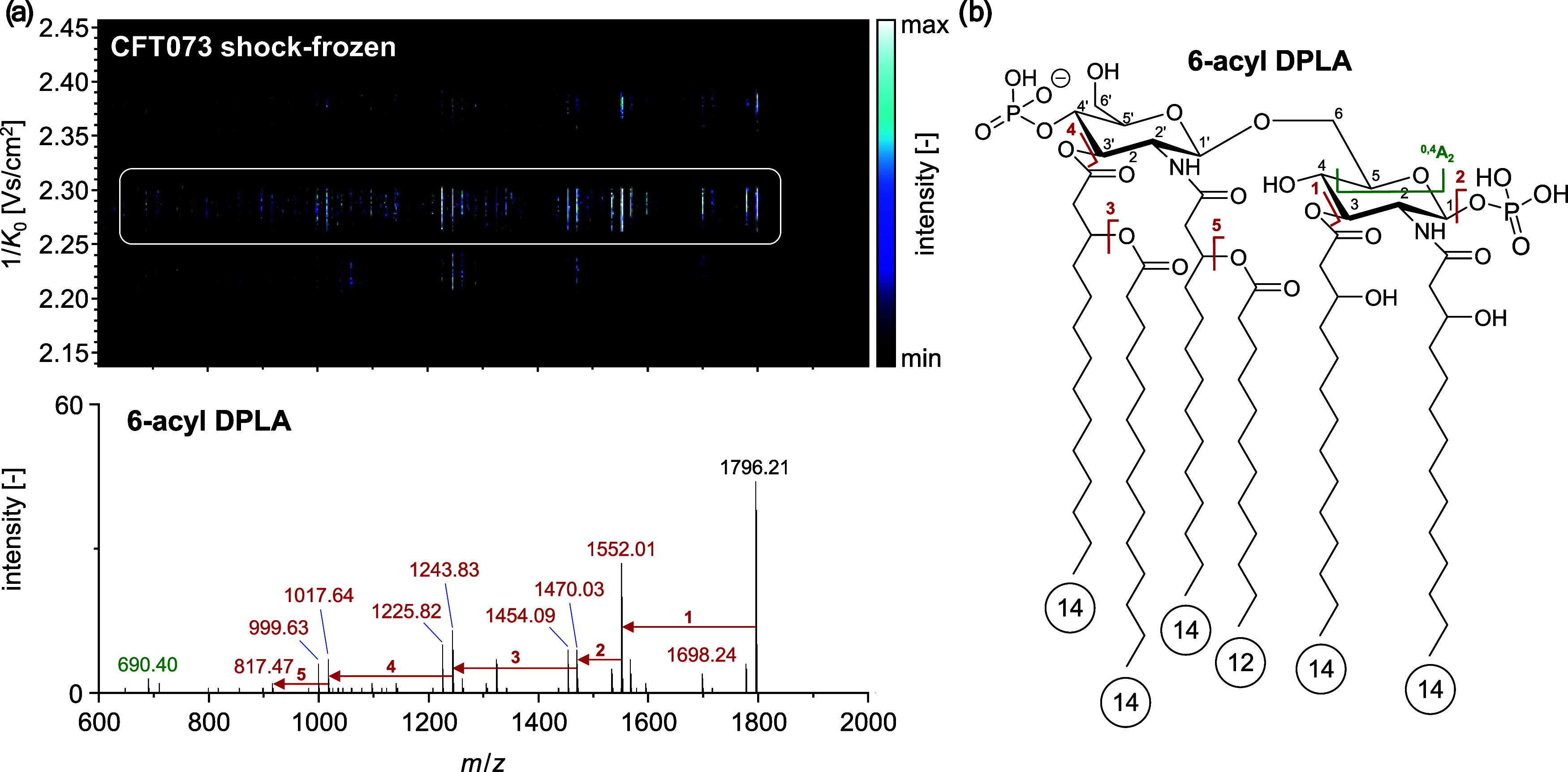
(a) Heatmap of multiple mobility-resolved precursor fragments in shock-frozen *E. coli* CFT073 using MALDI-TIMS-MS/MS via prm-PASEF in negative ionization mode. The fragmentation of 6-acyl DPLA is highlighted, and the combined MS/MS spectrum for varying collision energies (90, 100, and 110 eV) is plotted below, as automated by the SIMSEF workflow. (b) Molecular structure of 6-acyl DPLA including fracture points in MS/MS.

Moreover, TIMS can be utilized for mobility-resolved fragmentation via PASEF. Recently, prm-PASEF was introduced as part of the SIMSEF workflow in mzmine for fast and straightforward fragmentation in imaging.^44^ The present study focuses on the transfer of prm-PASEF and SIMSEF for MALDI spot analysis. prm-PASEF provides high sensitivity in generating MS/MS spectra^40,41^ and offers the additional benefit of a faster speed than conventional MS/MS experiments in MALDI spot analysis based on the mobility-resolved fragmentation. More precisely, prm-PASEF allows for the fragmentation of multiple compounds with varying mobility values in a single acquisition. In Figure 2, the prm-PASEF fragmentation of multiple lipid A species in negative ionization mode is illustrated, and the SIMSEF spectrum of 6-acyl DPLA is highlighted. As fragmentation occurs subsequent to the mobility detection, the fragments are identified horizontally to the corresponding precursor, showing lower *m*/*z* values, highlighted by a white rectangular box. The MS/MS spectrum for 6-acyl DPLA is obtained by combining spectra for multiple collision energies in mzmine using the SIMSEF workflow. This results in clean MS/MS spectra with maximum amount of information for lipid annotation. In more detail, the SIMSEF workflow allowed for a rapid characterization of lipid A compounds by the use of prm-PASEF within the mzmine software solution. In this software, suitable precursors for fragmentation were directly predefined by means of MS1 library-based lipid annotation. This resulted in a spot-dependent precursor list for semitargeted lipid A profiling in MALDI-TIMS-MS/MS analysis. The precursors were subsequently ordered to minimize acquisition time and fragmented in succession using multiple collision energies on a randomly moving sample spot, which usually only few minutes per spot. This workflow provides automated MS/MS-based MALDI profiling for structural elucidation, which is usually a manual and laborious process in a faster way than conventional LC-MS/MS-based lipid A profiling.

Moreover, mobility-resolved fragmentation has the advantage of producing clean spectra for isobaric and isomeric compounds with differences in CCS values. Typical isomers in lipid A analysis include regioisomeric MPLA with a varying position of the phosphate headgroup. The phosphate headgroup can be attached at the C1 or at the C4′ sugar position (hereafter referred to as C1P and C4′P, respectively). The C1P and C4’P MPLA regioisomers can be separated by means of chromatography^10^ or electrophoresis.^18,19^ However, these techniques cannot be used in conjunction with MALDI spot analysis. It is therefore of major interest to investigate the TIMS separation of regioisomeric MPLA.

**Figure 3:**
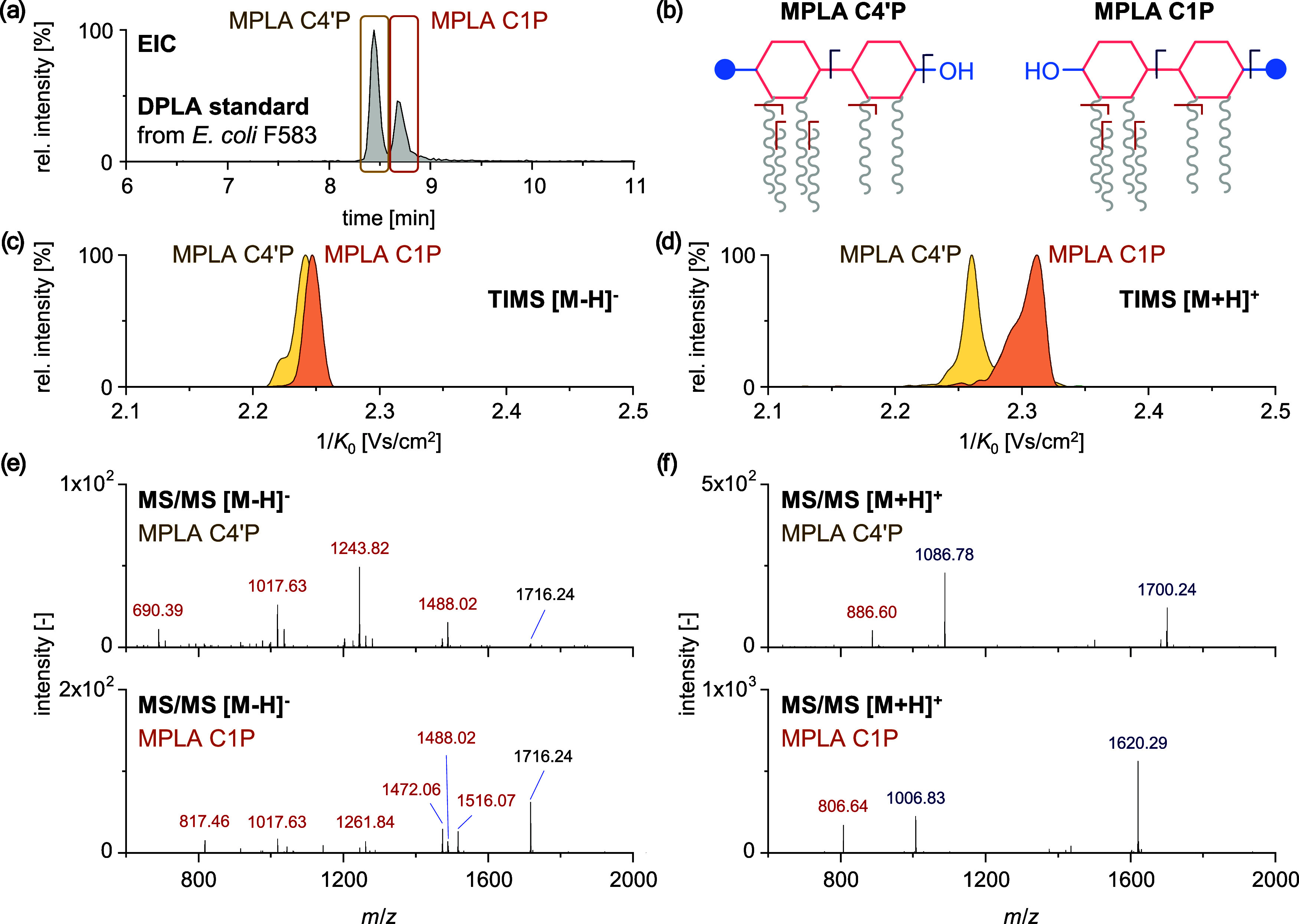
(a) EICs of regioisomeric 6-acyl MPLA C4′P and MPLA C1P in the DPLA standard extract from *E. coli* F583 using RP-HPLC-TIMS-MS/MS. (b) Schematic structure of these regioisomers (disaccharide backbone: red, fatty acyl groups: gray, phosphate head groups: blue). Mobilograms of MPLA C4′P and MPLA C1P in negative (c) and positive (d) ionization modes as well as (e, f) respective fragmentation spectra in both ionization modes (negative: 90 eV, positive: 50 eV, respectively).

The DPLA standard extract was subjected to examination for MPLA regioisomers using LC-TIMS-MS/MS (Figure 3). Therefore, the reversed-phase (RP)-HPLC method was optimized for accelerated lipid A analysis with tailored separation of regioisomeric MPLA based on the study conducted by Froning et al.^10^ (SI-6).

For 6-acyl MPLA, two isomeric compounds were chromatographically resolved. The mobility behavior of these regioisomers was characterized in both negative and positive ionization modes. In negative ionization mode, the mobility values observed for MPLA C4′P and MPLA C1P as deprotonated adducts were found to be similar (△CCS = 0.2%). Thus, the resolving power of TIMS is not sufficient to resolve these isomers. Furthermore, the fragmentation of both MPLA regioisomers is based on the neural loss of fatty acyl groups in the negative ionization mode. However, distinct differences were observed in the overall fragmentation pattern, indicating a consecutive fragmentation pattern for MPLA C4′P and a competitive pattern for MPLA C1P. These differences can provide a first indication of the phosphate position, but confirmation using specific fragments in positive ionization mode is required. Notably, in positive ionization mode, considerable differences in the CCS value of the protonated adducts of MPLA C4’P and MPLA C1P were observed (ΔCCS = 2.2%). This charge-induced change in the mobility separation was also observed for regioisomeric phospholipids in our recent study.^29^ Thus, the mobility separation enables direct analysis of regioisomeric MPLA using MALDI-TIMS-MS/MS. Furthermore, the positive ionization mode offers the advantage to produce specific fragments for MPLA regioisomers based on the fragmentation of the C1 and C1′ glycosidic bonds.^49^ Therefore, a neutral loss of H_2_O was observed for MPLA C4′P, while a unique loss of H_3_PO_4_ was detected for MPLA C1P. This results in a mass shift of △*m*/*z* 80. The same mass shift is also generated for the fragment of the C1′ glycosidic bonds of MPLA C4′P and MPLA C1P. The TIMS separation of regioisomeric lipid A species was also obtained for [M + Na]^+^ adducts in ESI and MALDI, as described thoroughly in the Supporting Information (SI-5).

### Automated MS/MS-Based Lipid A Annotation

In addition to the transfer of the SIMSEF workflow for MALDI spot analysis, this study aimed to develop an automated evaluation of the MALDI-TIMS-MS/MS data. A major challenge in this context is the automated annotation of lipid A species due to the huge diversity of potential lipid A species in combination with the large number of consecutive fragments. Typically, MS1 library-based lipid annotation as well as manual and laborious screening of MS/MS spectra is performed. In contrast, we used directly generated custom lipid classes in mzmine for the rapid and robust characterization of lipid A species.

Using the custom lipid class tool, a lipid A database was created, which included accurate mass, isotopic distribution, and fragmentation pattern for precise and automated annotation. The lipid A database has a modular structure based on the lipid backbone, side chain combinations, and individual fragmentation patterns. For instance, MPLA have a C_12_H_23_O_12_P_1_ lipid backbone formula and DPLA C_12_H_24_O_15_P_2_ (H_1_O_3_P_1_ backbone difference, △*m*/*z* 80). Using that information, the lipid backbone is extendable to different modifications, including PEtN or AraN (PEtN: C_2_H_6_N_1_O_3_P_1_ backbone difference, △*m*/*z* 123; AraN: C_5_H_9_N_1_O_3_, △*m*/*z* 131). Furthermore, the number and type of side chains attached to the lipid backbone are specified, leading to a huge variety of custom lipid A classes ranging from two to seven acylations. Each lipid A class is based on two amide mono hydroxy chains. Additionally, the degree of acylation determines whether acyl mono hydroxy chains as primary or acyl chains as secondary are attached. For instance, 6-acyl MPLA (2 sec) are customized based on the MPLA backbone and six side chains, including 2× amide mono hydroxy chains, 2× acyl mono hydroxy chains (2× primary), and 2× acyl chains (2× secondary).

In order to reduce the number of false positives in comparison with MS1-based annotation, fragmentation rules were defined for MS2-based lipid annotation. The high number of potential consecutive neutral loss fragments in the lipid A fragmentation pattern results in a vast number of fragmentation combination options, which presents a significant challenge for automated MS2-based lipid annotation. However, even if no comprehensive representation of the fragmentation pattern is provided, it is possible to achieve satisfactory results in the lipid annotation. In negative ionization mode, the introduced fragmentation rules for lipid A included the precursor ion and the neutral loss of one acyl chain fragment in negative ionization mode (for DPLA: the neutral loss of the H_3_PO_4_ headgroup was additionally defined). In contrast, in positive ionization mode, the precursor ion and the neutral loss of the C1 glycosidic bond were defined (neutral loss of the H_3_PO_4_ headgroup in the case of C1P MPLA and DPLA, or of the H_2_O headgroup in the case of C4′P MPLA). For modified lipid A, neutral losses of the headgroup modifications were added to the fragmentation rules for both ionization modes.

In total, 72 custom lipid A classes covering 2832 lipid species (C10–C16 side chains, no double bonds) were defined for this study including MPLA and DPLA with potential modifications (PEtN, AraN) and varying acylation (two acyl no secondary, three acyl 0–1 secondary, four acyl 0–2 secondary, five acyl 1–2 secondary, six acyl 2–3 secondary, seven acyl 3 secondary, eight acyl 4 secondary). In positive ionization mode, additional custom lipid A classes were developed for the differentiation of C1P MPLA and C4′P MPLA based on their respective fragmentation pattern. This resulted in the creation of a straightforward lipid A annotation, which serves to minimize both the number of false positives and the amount of time required for the manual verification process. The annotation process in mzmine took less than a second. The custom lipid A classes developed are provided in Table S2 of the Supporting Information and as JSON files (doi: 10.25345/C5707X11F).

### Sample Preparation Effect on the Lipid A Profile

**Figure 4:**
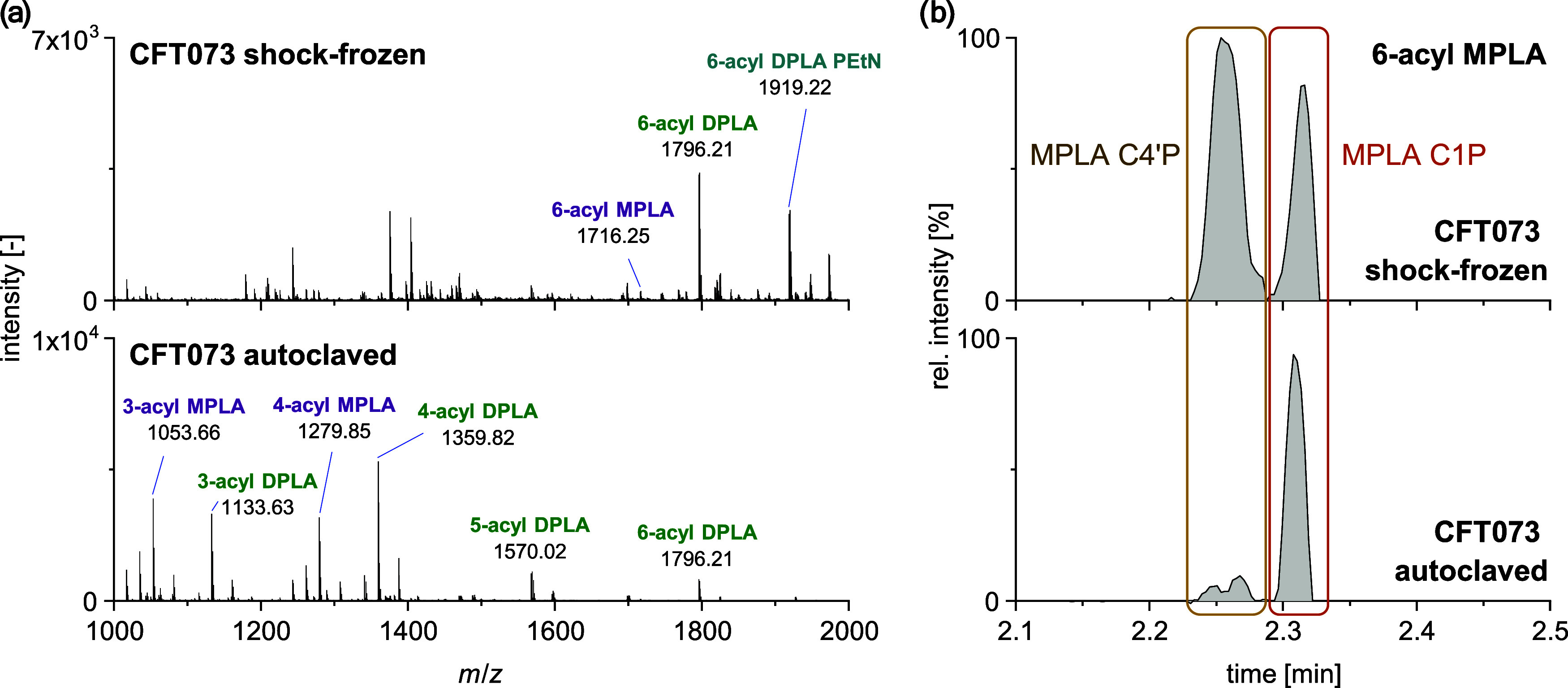
Influence of the sample preparation on (a) the lipid A profile in general and (b) the phosphate position of 6-acyl MPLA species in shock-frozen (top) and autoclaved (bottom) *E. coli* CFT073 using MALDI-TIMS-MS/MS. MPLA, DPLA, and modifications are color-coded.

Using the developed MALDI-TIMS-MS/MS workflow, we investigated the influence of different sample preparation procedures on the lipid A profile of *E. coli* CFT073. Therefore, a batch of *E. coli* bacteria was shock-frozen and treated with organic solvents to ensure cell death, whereas other bacteria were additionally autoclaved. For confirmation, the samples were analyzed by LC-TIMS-MS/MS as well. The MALDI-TIMS-MS/MS method showed improved performance in the identification of DPLA species (SI-7: Detailed comparison including literature). In Figure 4a, the MS spectra obtained for shock-frozen and autoclaved *E. coli* CFT073 in negative ionization mode are illustrated. Lipid A species in shock-frozen *E. coli* CFT073 were primarily 6-acylated, while less acylated species were detected in the autoclaved sample (SI-8). In more detail, lipid A species ranging from 3-acyl to 6-acyl were observed in autoclaved *E. coli* CFT073, with the main species being 3-acylated and 4-acylated. Therefore, the autoclaving process may have resulted in the degradation of lipid A molecules, leading to the loss of the fatty acyl groups that are bound to the disaccharide backbone. Moreover, TIMS in conjunction with mobility-resolved fragmentation spectra in positive ionization mode revealed a combination of C1P and C4′P phosphorylation sites of 6-acyl MPLA in shock-frozen *E. coli* CFT073. In contrast, autoclaved *E. coli* CFT073 exhibited phosphorylation exclusively on the C1P position (Figure 4b). Additionally, for the less acylated lipid A species in autoclaved *E. coli* CFT073, only C1P MPLA species were observed. This may indicate potential degradation of the phosphorylated C4′P headgroup by the autoclavation process. Similar results were obtained in LC-TIMS-MS/MS (SI-9), confirming the degradation of lipid A in autoclaved *E. coli* CFT073.

**Figure 5:**
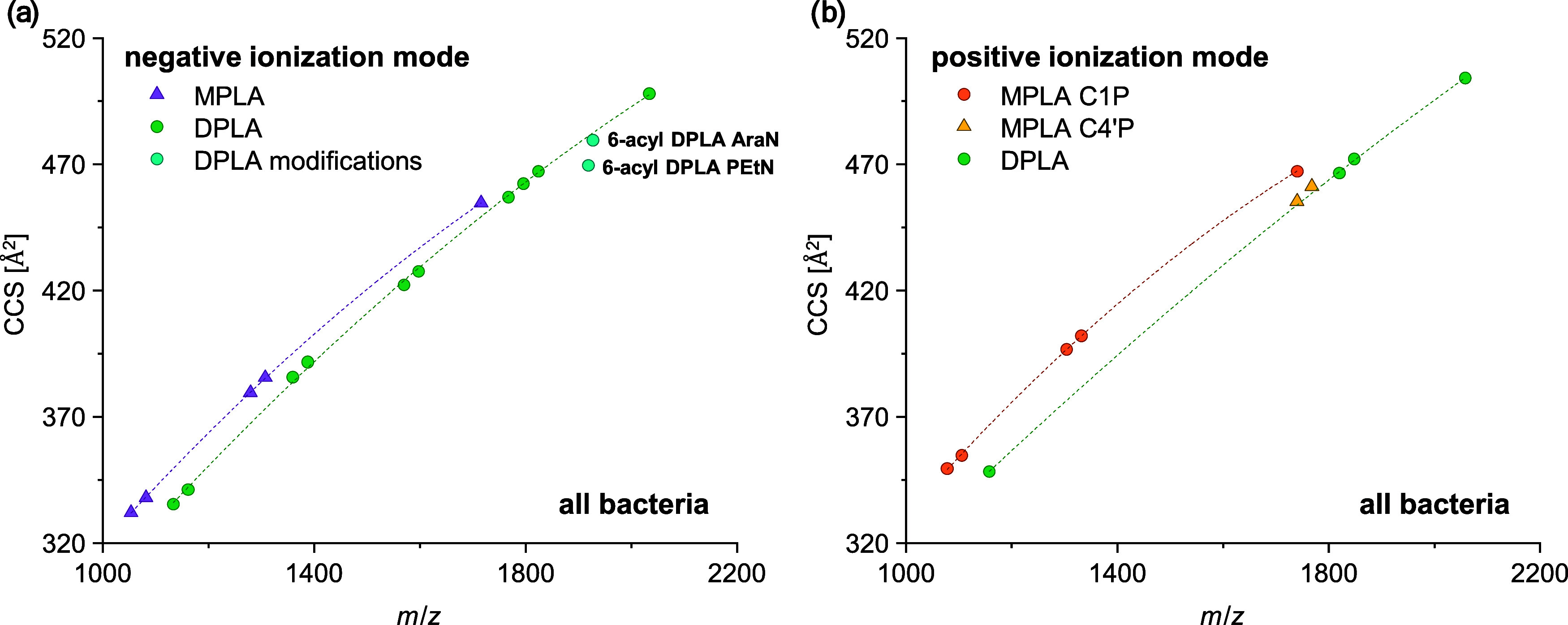
IM-MS plots of the identified lipid A species in both autoclaved and shock-frozen *E. coli* CFT073 using MALDI-TIMS-MS/MS in (a) negative and (b) positive ionization modes.

The gradual degradation of *E. coli* CFT073 by autoclaving has the benefit that a considerable number of diverse lipid A species were identified. This can be utilized as a first step to develop a CCS database, which might reveal the impact of varying building blocks on the three-dimensional structure of lipid A. The identified lipid A species in both autoclaved *E. coli* CFT073 and shock-frozen *E. coli* CFT073 in positive and negative ionization modes are illustrated in the IM-MS plots in Figure 5. In negative ionization mode, 17 lipid A species (5 MPLA, 10 DPLA, and 2 modified DPLA species) were identified, while 11 species (5 MPLA C1P, 2 MPLA C4′P, and 4 DPLA) were further characterized on the molecular species level in positive ionization mode. The lipid A species were detected as [M – H]^−^ and [M + Na]^+^ adducts in negative and positive ionization mode, respectively. Detailed side chain information on the identified lipid A species is provided in table format in the Supporting Information (SI-7).

The IM-MS plot in negative ionization mode confirmed different trajectories for MPLA and DPLA over various degrees of acylation, ranging from 3-acyl to 7-acyl lipid A species. Please note that the generated database comprised up to 8 acylations to even cover this uncommon lipid A structures. Moreover, the PEtN and AraN modifications of the predominant 6-acyl DPLA species in shock-frozen *E. coli* CFT073 demonstrated a shift in the DPLA trajectory. A stronger shift was observed for PEtN modifications, which showed a relatively low impact on the CCS value of lipid A. Additionally, the MPLA regioisomers could be subjected to further comparison through the IM-MS plot in positive ionization mode. This plot revealed different trajectories for MPLA C1P and C4′P. Notably, the mass/mobility of MPLA C4′P species were similar to the trajectory of DPLA. Moreover, the trajectory for MPLA C1P showed a higher impact of C1P phosphorylation site on the CCS value compared to C4’P. These CCS relationships can help tremendously in future mobility-enhanced lipid A applications, i.e., by contribution in the development of *in-silico* CCS databases based on the results obtained for the lipid A profile of autoclaved and shock-frozen *E. coli* CFT073.

## Conclusions

Current methods for lipid A analysis are either time-consuming and laborious or show only minor sensitivity. In this study, we developed a MALDI-TIMS-MS/MS-based workflow in conjunction with a microextraction, a data set-dependent fragmentation strategy, and a software-driven annotation for efficient and automated bacterial screening in MALDI spot analysis.

The developed tube-based microextraction for dried-droplet MALDI spot analysis provided an efficient sample preparation with enhanced sensitivity for lipid A analysis. Sensitivity was further improved by introducing TIMS as an additional dimension. Next to the sensitivity enhancement, the integrated microextraction served to ensure reliable sample homogeneity in the MALDI application. This facilitated the production of clean MS/MS spectra via mobility-resolved prm-PASEF and enabled automated MS/MS-based lipid A profiling by adapting the SIMSEF strategy for MALDI spot analysis on a randomly moving sample spot. Using this adaptation, lipid A precursors were fragmented spot-dependently with multiple collision energies, thereby maximizing the amount of information for structural elucidation.

Moreover, data evaluation was facilitated in the mzmine software solution by introducing custom lipid A classes for robust annotation based on the first neutral loss obtained in lipid A fragmentation spectra. This template may be extended to further lipid classes, such as for modified lipid A. The developed custom lipid class tool can also be used for rapid lipid database building and subsequent annotation of less covered lipid classes in the literature as well as for modified lipids, making it also interesting for epilipidomics research.

In combination with the enhanced selectivity of TIMS in positive ionization mode, a regioisomer-specific annotation of the MPLA species was achieved. Furthermore, CCS values for a highly confident annotation were introduced, and the impact of varying building blocks on the three-dimensional structure of lipid A was revealed. Our developed workflow may be used in future studies for efficient and user-friendly bacterial screening applications, comprising lipid A analysis or other relevant biomarkers in bacteria.

## Supporting Information Available

(SI-1) Cultivation, (SI-2) sample preparation, (SI-3) instrumental setup, (SI-4) data processing, (SI-5) MALDI-TIMS-MS/MS method development, (SI-6) LC-TIMS-MS/MS method development, (SI-7) comparison of identified lipid A species, (SI-8) identified lipid A species by MALDI-TIMS-MS/MS, (SI-9) identified lipid A species by LC-TIMS-MS/MS, (SI-10) references. Raw data, mzbatch files, and custom lipid classes JSON files are publicly available on MassIVE MSV000096293 (doi:10.25345/C5707X11F). The developed mzmine custom lipid classes annotation tool is further explained in detail in the mzmine online documentation (https://mzmine.github.io/mzmine_documentation/module_docs/id_lipid_annotation/lipid-annotation.html#custom-lipid-classes-and-fragmentation-rules) and the source-code is also publicly available on GitHub (https://github.com/mzmine/mzmine) (PDF)

## References

[ref1] Erridge C.; Bennett-Guerrero E.; Poxton I. R. Structure and function of lipopolysaccharides. Microbes Infect. 2002, 4, 837–851. 10.1016/S1286-4579(02)01604-0. 12270731

[ref2] Park B. S.; Song D. H.; Kim H. M.; Choi B.-S.; Lee H.; Lee J.-O. The structural basis of lipopolysaccharide recognition by the TLR4-MD-2 complex. Nature 2009, 458, 1191–1195. 10.1038/nature07830. 19252480

[ref3] Rietschel E. T.; Kirikae T.; Schade F. U.; Mamat U.; Schmidt G.; Loppnow H.; Ulmer A. J.; Zähringer U.; Seydel U.; Di Padova F. Bacterial endotoxin: molecular relationships of structure to activity and function. FASEB J. 1994, 8, 217–225. 10.1096/fasebj.8.2.8119492. 8119492

[ref4] Matsuura M. Structural Modifications of Bacterial Lipopolysaccharide that Facilitate Gram-Negative Bacteria Evasion of Host Innate Immunity. Front. Immunol. 2013, 4, 109 10.3389/fimmu.2013.00109. 23745121 PMC3662973

[ref5] Raetz C. R. H.; Reynolds C. M.; Trent M. S.; Bishop R. E. Lipid A modification systems in gram-negative bacteria. Annu. Rev. Biochem. 2007, 76, 295–329. 10.1146/annurev.biochem.76.010307.145803. 17362200 PMC2569861

[ref6] Scott A. J.; Oyler B. L.; Goodlett D. R.; Ernst R. K. Lipid A structural modifications in extreme conditions and identification of unique modifying enzymes to define the Toll-like receptor 4 structure-activity relationship. Biochim. Biophys. Acta, Mol. Cell Biol. Lipids 2017, 1862, 1439–1450. 10.1016/j.bbalip.2017.01.004. 28108356 PMC5513793

[ref7] Steimle A.; Autenrieth I. B.; Frick J.-S. Structure and function: Lipid A modifications in commensals and pathogens. Int. J. Med. Microbiol. 2016, 306, 290–301. 10.1016/j.ijmm.2016.03.001. 27009633

[ref8] Ernst R. K.; Yi E. C.; Guo L.; Lim K. B.; Burns J. L.; Hackett M.; Miller S. I. Specific lipopolysaccharide found in cystic fibrosis airway *Pseudomonas aeruginosa*. Science 1999, 286, 1561–1565. 10.1126/science.286.5444.1561. 10567263

[ref9] Imoto M.; Kusumoto S.; Shiba T.; Naoki H.; Iwashita T.; Rietschel E. T.; Wollenweber H.-W.; Galanos C.; Lüderitz O. Chemical structure of *E. coli* lipid A: linkage site of acyl groups in the disaccharide backbone. Tetrahedron lett. 1983, 24, 4017–4020. 10.1016/S0040-4039(00)88251-9.

[ref10] Froning M.; Helmer P. O.; Hayen H. Identification and structural characterization of lipid A from *Escherichia coli*, *Pseudomonas putida* and *Pseudomonas taiwanensis* using liquid chromatography coupled to high-resolution tandem mass spectrometry. Rapid Commun. Mass Spectrom. 2020, 34, e8897 10.1002/rcm.8897. 32673427

[ref11] Sándor V.; Dörnyei Á.; Makszin L.; Kilár F.; Péterfi Z.; Kocsis B.; Kilár A. Characterization of complex, heterogeneous lipid A samples using HPLC-MS/MS technique I. Overall analysis with respect to acylation, phosphorylation and isobaric distribution. J. Mass Spectrom. 2016, 51, 1043–1063. 10.1002/jms.3839. 27506631

[ref12] Guan X. L.; Loh J. Y.-X.; Lizwan M.; Chan S. C. M.; Kwan J. M. C.; Lim T. P.; Koh T. H.; Hsu L.-Y.; Lee B. T. K. LipidA-IDER to Explore the Global Lipid A Repertoire of Drug-Resistant Gram-Negative Bacteria. Anal. Chem. 2023, 95, 602–611. 10.1021/acs.analchem.1c03566. 36599414 PMC9850412

[ref13] Okahashi N.; Ueda M.; Matsuda F.; Arita M. Analyses of Lipid A Diversity in Gram-Negative Intestinal Bacteria Using Liquid Chromatography-Quadrupole Time-of-Flight Mass Spectrometry. Metabolites 2021, 11, 197 10.3390/metabo11040197. 33810392 PMC8065654

[ref14] Larrouy-Maumus G.; Clements A.; Filloux A.; McCarthy R. R.; Mostowy S. Direct detection of lipid A on intact Gram-negative bacteria by MALDI-TOF mass spectrometry. J. Microbiol. Methods 2016, 120, 68–71. 10.1016/j.mimet.2015.12.004. 26656001 PMC4717120

[ref15] Yang H.; Chandler C. E.; Jackson S. N.; Woods A. S.; Goodlett D. R.; Ernst R. K.; Scott A. J. On-Tissue Derivatization of Lipopolysaccharide for Detection of Lipid A Using MALDI-MSI. Anal. Chem. 2020, 92, 13667–13671. 10.1021/acs.analchem.0c02566. 32902263 PMC8717242

[ref16] Yang H.; Smith R. D.; Chandler C. E.; Johnson J. K.; Jackson S. N.; Woods A. S.; Scott A. J.; Goodlett D. R.; Ernst R. K. Lipid A Structural Determination from a Single Colony. Anal. Chem. 2022, 94, 7460–7465. 10.1021/acs.analchem.1c05394. 35576511 PMC9392460

[ref17] Sorensen M.; Chandler C. E.; Gardner F. M.; Ramadan S.; Khot P. D.; Leung L. M.; Farrance C. E.; Goodlett D. R.; Ernst R. K.; Nilsson E. Rapid microbial identification and colistin resistance detection via MALDI-TOF MS using a novel on-target extraction of membrane lipids. Sci. Rep. 2020, 10, 21536 10.1038/s41598-020-78401-3. 33299017 PMC7725828

[ref18] Sándor V.; Berkics B. V.; Kilár A.; Kocsis B.; Kilár F.; Dörnyei Á. NACE-ESI-MS/MS method for separation and characterization of phosphorylation and acylation isomers of lipid A. Electrophoresis 2020, 41, 1178–1188. 10.1002/elps.201900251. 32335940

[ref19] Sándor V.; Űrmös B.; Aissa I.; Dörnyei Á.; Kilár A. Characterization of isomeric lipid A species from *Pseudomonas aeruginosa* PAO1 by non-aqueous capillary electrophoresis with positive and negative ion electrospray tandem mass spectrometry. Arab. J. Chem. 2023, 16, 104944 10.1016/j.arabjc.2023.104944.

[ref20] Chen Y.; Lehotay S. J.; Moreau R. A. Supercritical fluid chromatography-tandem mass spectrometry for the analysis of lipid A. Anal. Methods 2013, 5, 6864–6869. 10.1039/c3ay41344f.

[ref21] Henderson J. C.; O’Brien J. P.; Brodbelt J. S.; Trent M. S. Isolation and chemical characterization of lipid A from gram-negative bacteria. J. Vis. Exp. 2013, 16, 50623 10.3791/50623. PMC3885993 24084191

[ref22] Dodds J. N.; Baker E. S. Ion Mobility Spectrometry: Fundamental Concepts, Instrumentation, Applications, and the Road Ahead. J. Am. Soc. Mass Spectrom. 2019, 30, 2185–2195. 10.1007/s13361-019-02288-2. 31493234 PMC6832852

[ref23] Ridgeway M. E.; Lubeck M.; Jordens J.; Mann M.; Park M. A. Trapped ion mobility spectrometry: A short review. Int. J. Mass Spectrom. 2018, 425, 22–35. 10.1016/j.ijms.2018.01.006.

[ref24] Gabelica V.; Marklund E. Fundamentals of ion mobility spectrometry. Curr. Opin. Chem. Biol. 2018, 42, 51–59. 10.1016/j.cbpa.2017.10.022. 29154177

[ref25] Leaptrot K. L.; May J. C.; Dodds J. N.; McLean J. A. Ion mobility conformational lipid atlas for high confidence lipidomics. Nat. Commun. 2019, 10, 985 10.1038/s41467-019-08897-5. 30816114 PMC6395675

[ref26] Paglia G.; Kliman M.; Claude E.; Geromanos S.; Astarita G. Applications of ion-mobility mass spectrometry for lipid analysis. Anal. Bioanal. Chem. 2015, 407, 4995–5007. 10.1007/s00216-015-8664-8. 25893801

[ref27] Zhou Z.; Tu J.; Xiong X.; Shen X.; Zhu Z.-J. LipidCCS: Prediction of Collision Cross-Section Values for Lipids with High Precision To Support Ion Mobility-Mass Spectrometry-Based Lipidomics. Anal. Chem. 2017, 89, 9559–9566. 10.1021/acs.analchem.7b02625. 28764323

[ref28] Ross D. H.; Cho J. H.; Zhang R.; Hines K. M.; Xu L. LiPydomics: A Python Package for Comprehensive Prediction of Lipid Collision Cross Sections and Retention Times and Analysis of Ion Mobility-Mass Spectrometry-Based Lipidomics Data. Anal. Chem. 2020, 92, 14967–14975. 10.1021/acs.analchem.0c02560. 33119270 PMC7816765

[ref29] Rudt E.; Schneider S.; Hayen H. Hyphenation of Liquid Chromatography and Trapped Ion Mobility - Mass Spectrometry for Characterization of Isomeric Phosphatidylethanolamines with Focus on *N*-Acylated Species. J. Am. Soc. Mass Spectrom. 2024, 35, 1584–1593. 10.1021/jasms.4c00162. 38842006

[ref30] Helmer P. O.; Behrens A.; Rudt E.; Karst U.; Hayen H. Hydroperoxylated vs Dihydroxylated Lipids: Differentiation of Isomeric Cardiolipin Oxidation Products by Multidimensional Separation Techniques. Anal. Chem. 2020, 92, 12010–12016. 10.1021/acs.analchem.0c02605. 32867498

[ref31] Scholz J.; Rudt E.; Gremme A.; Gaßmöller C. M.; Bornhorst J.; Hayen H. Hyphenation of supercritical fluid chromatography and trapped ion mobility-mass spectrometry for quantitative lipidomics. Anal. Chim. Acta 2024, 1317, 342913 10.1016/j.aca.2024.342913. 39030025

[ref32] Kirkwood K. I.; Pratt B. S.; Shulman N.; Tamura K.; MacCoss M. J.; MacLean B. X.; Baker E. S. Utilizing Skyline to analyze lipidomics data containing liquid chromatography, ion mobility spectrometry and mass spectrometry dimensions. Nat. Protoc. 2022, 17, 2415–2430. 10.1038/s41596-022-00714-6. 35831612 PMC9633456

[ref33] Sans M.; Feider C. L.; Eberlin L. S. Advances in mass spectrometry imaging coupled to ion mobility spectrometry for enhanced imaging of biological tissues. Curr. Opin. Chem. Biol. 2018, 42, 138–146. 10.1016/j.cbpa.2017.12.005. 29275246 PMC5828985

[ref34] Rivera E. S.; Djambazova K. V.; Neumann E. K.; Caprioli R. M.; Spraggins J. M. Integrating ion mobility and imaging mass spectrometry for comprehensive analysis of biological tissues: A brief review and perspective. Biol. Mass Spectrom. 2020, 55, e4614 10.1002/jms.4614. PMC8211109 32955134

[ref35] Helmer P. O.; Nordhorn I. D.; Korf A.; Behrens A.; Buchholz R.; Zubeil F.; Karst U.; Hayen H. Complementing Matrix-Assisted Laser Desorption Ionization-Mass Spectrometry Imaging with Chromatography Data for Improved Assignment of Isobaric and Isomeric Phospholipids Utilizing Trapped Ion Mobility-Mass Spectrometry. Anal. Chem. 2021, 93, 2135–2143. 10.1021/acs.analchem.0c03942. 33416303

[ref36] Djambazova K. V.; Klein D. R.; Migas L. G.; Neumann E. K.; Rivera E. S.; van de Plas R.; Caprioli R. M.; Spraggins J. M. Resolving the Complexity of Spatial Lipidomics Using MALDI TIMS Imaging Mass Spectrometry. Anal. Chem. 2020, 92, 13290–13297. 10.1021/acs.analchem.0c02520. 32808523

[ref37] Kyle J. E.; Zhang X.; Weitz K. K.; Monroe M. E.; Ibrahim Y. M.; Moore R. J.; Cha J.; Sun X.; Lovelace E. S.; Wagoner J.; Polyak S. J.; Metz T. O.; Dey S. K.; Smith R. D.; Burnum-Johnson K. E.; Baker E. S. Uncovering biologically significant lipid isomers with liquid chromatography, ion mobility spectrometry and mass spectrometry. Analyst 2016, 141, 1649–1659. 10.1039/C5AN02062J. 26734689 PMC4764491

[ref38] Meier F.; Beck S.; Grassl N.; Lubeck M.; Park M. A.; Raether O.; Mann M. Parallel Accumulation-Serial Fragmentation (PASEF): Multiplying Sequencing Speed and Sensitivity by Synchronized Scans in a Trapped Ion Mobility Device. J. Proteome Res. 2015, 14, 5378–5387. 10.1021/acs.jproteome.5b00932. 26538118

[ref39] Meier F.; Brunner A.-D.; Koch S.; Koch H.; Lubeck M.; Krause M.; Goedecke N.; Decker J.; Kosinski T.; Park M. A.; Bache N.; Hoerning O.; Cox J.; Räther O.; Mann M. Online Parallel Accumulation-Serial Fragmentation (PASEF) with a Novel Trapped Ion Mobility Mass Spectrometer. Mol. Cell. Proteomics 2018, 17, 2534–2545. 10.1074/mcp.TIR118.000900. 30385480 PMC6283298

[ref40] Lesur A.; Schmit P.-O.; Bernardin F.; Letellier E.; Brehmer S.; Decker J.; Dittmar G. Highly Multiplexed Targeted Proteomics Acquisition on a TIMS-QTOF. Anal. Chem. 2021, 93, 1383–1392. 10.1021/acs.analchem.0c03180. 33331761

[ref41] Rudt E.; Feldhaus M.; Margraf C. G.; Schlehuber S.; Schubert A.; Heuckeroth S.; Karst U.; Jeck V.; Meyer S. W.; Korf A.; Hayen H. Comparison of Data-Dependent Acquisition, Data-Independent Acquisition, and Parallel Reaction Monitoring in Trapped Ion Mobility Spectrometry-Time-of-Flight Tandem Mass Spectrometry-Based Lipidomics. Anal. Chem. 2023, 95, 9488–9496. 10.1021/acs.analchem.3c00440. 37307407

[ref42] Wolf C.; Behrens A.; Brungs C.; Mende E. D.; Lenz M.; Piechutta P. C.; Roblick C.; Karst U. Mobility-resolved broadband dissociation and parallel reaction monitoring for laser desorption/ionization-mass spectrometry-Tattoo pigment identification supported by trapped ion mobility spectrometry. Anal. Chim. Acta 2023, 1242, 340796 10.1016/j.aca.2023.340796. 36657890

[ref43] Gruber L.; Schmidt S.; Enzlein T.; Vo H. G.; Cairns J. L.; Ucal Y.; Keller F.; Abu Sammour D.; Rudolf R.; Eckhardt M.; Iakab S. A.; Bindila L.; Hopf C. Deep MALDI-MS Spatial ‘Omics guided by Quantum Cascade Laser Mid-infrared Imaging Microscopy bioRxiv 2023. 10.1038/s41467-025-59839-3 PMC12098849 40404613

[ref44] Heuckeroth S.; Behrens A.; Wolf C.; Fütterer A.; Nordhorn I. D.; Kronenberg K.; Brungs C.; Korf A.; Richter H.; Jeibmann A.; Karst U.; Schmid R. On-tissue dataset-dependent MALDI-TIMS-MS2 bioimaging. Nat. Commun. 2023, 14, 7495 10.1038/s41467-023-43298-9. 37980348 PMC10657435

[ref45] Mobley H. L.; Green D. M.; Trifillis A. L.; Johnson D. E.; Chippendale G. R.; Lockatell C. V.; Jones B. D.; Warren J. W. Pyelonephritogenic Escherichia coli and killing of cultured human renal proximal tubular epithelial cells: role of hemolysin in some strains. Infect. Immun. 1990, 58, 1281–1289. 10.1128/iai.58.5.1281-1289.1990. 2182540 PMC258621

[ref46] Schmid R.; Heuckeroth S.; Korf A.; et al. Integrative analysis of multimodal mass spectrometry data in MZmine 3. Nat. Biotechnol. 2023, 41, 447–449. 10.1038/s41587-023-01690-2. 36859716 PMC10496610

[ref47] Lerner R.; Baker D.; Schwitter C.; Neuhaus S.; Hauptmann T.; Post J. M.; Kramer S.; Bindila L. Four-dimensional trapped ion mobility spectrometry lipidomics for high throughput clinical profiling of human blood samples. Nat. Commun. 2023, 14, 937 10.1038/s41467-023-36520-1. 36806650 PMC9941096

[ref48] Tsugawa H.; Ikeda K.; Takahashi M.; Satoh A.; Mori Y.; Uchino H.; Okahashi N.; Yamada Y.; Tada I.; Bonini P.; Higashi Y.; Okazaki Y.; Zhou Z.; Zhu Z.-J.; Koelmel J.; Cajka T.; Fiehn O.; Saito K.; Arita M.; Arita M. A lipidome atlas in MS-DIAL 4. Nat. Biotechnol. 2020, 38, 1159–1163. 10.1038/s41587-020-0531-2. 32541957

[ref49] Sándor V.; Kilár A.; Kilár F.; Kocsis B.; Dörnyei Á. Characterization of complex, heterogeneous lipid A samples using HPLC-MS/MS technique III. Positive-ion mode tandem mass spectrometry to reveal phosphorylation and acylation patterns of lipid A. J. Mass Spectrom. 2018, 53, 146–161. 10.1002/jms.4046. 29144587

