# Supplementary material for: Rapid MALDI-MS/MS-Based Profiling of Lipid A Species from Gram-Negative Bacteria Utilizing Trapped Ion Mobility Spectrometry and mzmine
Source: Anal Chem. 2025 Apr 1;97(14):7781–8. doi: 10.1021/acs.analchem.4c05989 (PMC12004357; doi:10.1021/acs.analchem.4c05989)
Supplement: Supplementary file 1 — ac4c05989_si_001.pdf [file ac4c05989_si_001.pdf]

# Supporting Information

## Rapid MALDI-MS/MS-based profiling of lipid A species from gram-negative bacteria utilizing trapped ion mobility spectrometry and mzmine

Edward Rudt[a], Matti Froning[a], Steffen Heuckeroth[b], Lucas Ortmann[a], Julia Diemand[a], Linus Hörnschemeyer[a], Alexander Pleger[a], Max Vinzelberg[a], Robin Schmid[b,c], Tomáš Pluskal[c], Ulrich Dobrindt[d], Heiko Hayen[a], Ansgar Korf[b,c*]

[a] *Institute of Inorganic and Analytical Chemistry, University of Münster, Corrensstraße 48, D-48149 Münster, Germany*

[b] *mzio GmbH, Altenwall 26, D-28195 Bremen, Germany*

[c] *Institute of Organic Chemistry and Biochemistry of the Czech Academy of Sciences, Prague, Czech Republic*

[d] *Institute of Hygiene, University of Münster, Mendelstraße 7, D-48149 Münster, Germany*

[*]Corresponding author:

Dr. Ansgar Korf

mzio GmbH, Altenwall 26, D-28195 Bremen, Germany

Tel: +49 176 47342320

Email: ansgar.korf@mzio.io

# Table of Contents

**SI-1: Cultivation** .... 2
**SI-2: Sample preparation** .... 3
**SI-3: Instrumental setup** .... 4
**SI-4: Data processing** .... 6
**SI-5: MALDI-TIMS-MS/MS method development** .... 13
**SI-6: LC-TIMS-MS/MS method development** .... 17
**SI-7: Comparison of identified lipid A species** .... 18
**SI-8: Identified lipid A species by MALDI-TIMS-MS/MS** .... 19
**SI-9: Identified lipid A species by LC-TIMS-MS/MS** .... 22
**SI-10: References** .... 24

## SI-1: Cultivation

*E. coli* CFT073 was cultivated at 37 °C in batch cultures in M9 minimal medium supplemented with 0.2% glucose for 16 hours and subsequently either shock-frozen or autoclaved additionally (three biological replicates each). The optical density ($OD_{600}$) was determined and is listed below.

**Table S1:** Overview of the $OD_{600}$ values observed for different biological replicates of *E. coli* CFT073, which were cultivated in M9 minimal medium and either shock-frozen or autoclaved.

| Biological replicate | $OD_{600}$ for shock-frozen | $OD_{600}$ for autoclaved |
|---|---|---|
| *E. coli* CFT073 M9a | 0.94 | 0.94 |
| *E. coli* CFT073 M9b | 0.97 | 0.97 |
| *E. coli* CFT073 M9c | 1.15 | 1.15 |

## SI-2: Sample preparation

Lipid A extraction for LC-MS/MS and MALDI-MS/MS was optimized based on modified protocols of Henderson *et al.*[1] and Sorensen *et al.*[2], respectively. The samples were treated identically, dried, and weighed, and then different protocols were carried out, as described in the following.

### General sample preparation

The cultivated bacteria were washed with 1 mL $H_2O$ and the supernatant removed after centrifugation (5 min, 10000 rpm). Then, the suspension was treated with 2 mL MeOH, 1 mL $CHCl_3$ and incubated for 1 h to ensure cell death. Afterwards, the samples were dried by several centrifugation steps (3x for 10 min, 5000 rpm each) including subsequent supernatant removing and evaporation using a gentle $N_2$ stream at 40 °C. The dried bacteria were weighed for subsequent sample preparation. A total amount of 50 mg bacteria was used for the microextraction prior to MALDI-MS/MS analysis.

### MALDI-MS/MS microextraction

Mild acid hydrolysis of lipid A from its oligosaccharide core was conducted by adding 200 µL citrate buffer (0.2 M citric acid and 0.1 M trisodium citrate) and heating for 1 h at 100 °C. Once cooled, the suspension was extracted by adding 400 µL $CHCl_3$/MeOH (1:1, *v*/*v*), followed by centrifugation (5 min, 5000 rpm). The resulting lower organic phase was then utilized directly for MALDI spot analysis. In more detail, 1 µL lipid A extract, followed by 1 µL matrix (negative ionization mode: 9-AA, positive ionization mode: FA; 10 mg/mL each in $CHCl_3$/MeOH (1:1, *v*/*v*)), were spotted on a MALDI target plate 384 ground steel (Bruker Daltonics GmbH & Co. KG, Bremen, Germany).

### LC-MS/MS extensive extraction

For extensive extraction prior to LC-MS/MS, 200 mg of the dried sample was further washed using 2x 4 mL $CHCl_3$/MeOH/PBS (1:2:0.8, *v*/*v*/*v*). Mild acid hydrolysis was conducted by adding 1.1 mL citrate buffer (0.2 M citric acid and 0.1 M trisodium citrate), sonicating the mixture, and heating it for 1 h at 100 °C. Once cooled, the suspension was extracted by adding 2.4 mL $CHCl_3$/MeOH (1:1, *v*/*v*), followed by centrifugation (10 min, 5000 rpm), and the collection of the lower organic phase. A two-phase mixture of $CHCl_3$/MeOH/$H_2O$ (2:1:1.8, *v*/*v*/*v*) was prepared for the following steps. The upper phase of the sample was reextracted using 1.2 mL of the lower phase of the aforementioned mixture. Afterwards, the lower phase of the sample was collected once more after centrifugation (10 min, 5000 rpm), and the combined phases were washed with 4.6 mL of the upper phase of the aforementioned $CHCl_3$/MeOH/$H_2O$ mixture. The sample extract was evaporated using a gentle $N_2$ stream at 40 °C and subsequently dissolved in 200 µL $CHCl_3$/MeOH (1:1, *v*/*v*).

## SI-3: Instrumental setup

MS measurements were performed on a timsTOF fleX instrument (Bruker Daltonics GmbH & Co. KG, Bremen, Germany), including an Apollo II source for ESI measurements and a Nd:YAG smartbeam 3D laser at 355 nm for MALDI. Measurements were conducted in both negative and positive ionization modes using either MALDI-TIMS-MS/MS or LC-TIMS-MS/MS. To provide the acquisition of high mobility ranges for TIMS analysis of lipid A, the source gas flow in the TIMS cartridge was adjusted. Thus, the vacuum of the system was changed to a pressure difference of 1.3 mbar between entrance and exit funnel. The following TIMS-MS parameters were utilized:

**Negative ionization mode:** Mass and mobility ranges were set as *m*/*z* 100 – 2300 and 1.50 – 2.50 Vs/cm$^2$ with 500 ms ramp time, 5.0 million ion charge control, 500 V endplate offset, 4 kV capillary voltage, 2.0 bar nebulizer gas ($N_2$), 9.0 L/min dry gas ($N_2$), 200°C drying temperature, 500.0 Vpp funnel 1 RF, 400.0 Vpp funnel 2 RF, 300.0 Vpp multipole RF, 100.0 V deflection delta, 5.0 eV quadrupole ion energy, 10.0 eV collision cell energy, 2500.0 Vpp collision cell RF, 100.0 µs transfer time, and 10.0 µs pre pulse storage. Tunnel voltages were set as: -20.0 V Δ1, -120.0 V Δ2, 80.0 V Δ3, 100.0 V Δ4, 0.0 V Δ5, 100.0 V Δ6, 220.0 V collision cell energy.

**Positive ionization mode:** Mass and mobility ranges were set as *m*/*z* 100 – 2300 and 1.50 – 2.65 Vs/cm$^2$ with 500 ms ramp time, 5.0 million ion charge control, 500 V endplate offset, -3.5 kV capillary voltage, 2.0 bar nebulizer gas ($N_2$), 9.0 L/min dry gas ($N_2$), 200°C drying temperature, 500.0 Vpp funnel 1 RF, 400.0 Vpp funnel 2 RF, 300.0 Vpp multipole RF, -100.0 V deflection delta, -5.0 eV quadrupole ion energy, -10.0 eV collision cell energy, 2500.0 Vpp collision cell RF, 100.0 µs transfer time, and 10.0 µs pre pulse storage. Tunnel voltages were set as: 20.0 V Δ1, 120.0 V Δ2, -80.0 V Δ3, -100.0 V Δ4, 0.0 V Δ5, -100.0 V Δ6, -220.0 V collision cell energy.

**Mass calibration:** A 5 mM sodium formate mixture in IPA/$H_2O$ (1:1, *v*/*v*) was used for mass calibration, and the ESI-L Low Concentration Tuning Mix (Agilent Technologies, Santa Clara, CA, USA) was utilized for mobility calibration (negative ionization mode: *m*/*z* 1333.9689 (1.393 Vs/cm$^2$), *m*/*z* 1633.9498 (1.568 Vs/cm$^2$), *m*/*z* 1933.9306 (1.741 Vs/cm$^2$); positive ionization mode: *m*/*z* 1221.9906 (1.407 Vs/cm$^2$), *m*/*z* 1521.9715 (1.407 Vs/cm$^2$), *m*/*z* 1821.9523 (1.407 Vs/cm$^2$)). To ensure high mobility accuracy, an online calibration segment was incorporated (13 – 15 min time range), as proposed by Helmer *et al.*[3] This involved the introduction of a 20 µL sample loop, filled with ESI-L Low Concentration Tuning Mix (0.5 µL/min flow rate), into the MS via valve switching.

**MALDI-MS:** Measurements were performed using 1000 laser shots (5x 200 shots) with 100 µm spot size and 1000 Hz frequency. In negative ionization mode, 60 % laser energy was used, and 70 % laser energy in positive ionization mode.

**MALDI-TIMS-MS/MS:** prm-PASEF was utilized via SIMSEF with 1.50 *m*/*z* isolation width and varying collision energies (negative ionization mode: 90 eV, 100 eV, 110 eV; positive ionization mode: 60 eV, 70 eV, 80 eV) for each precursor on each MALDI spot. Furthermore, fragmentation selection was limited to 0.02 – 0.04 Vs/cm$^2$ mobility window and to the top 40 annotated precursor, as identified through a preliminary MS1 library-based lipid annotation in mzmine.

**LC-MS:** Measurements were performed on a Vanquish Flex UHPLC system (Thermo Scientific, Dreieich, Germany), including a VF-A40-A dual split sampler FT, a VF-P32-A dual pump F, and a VH-C10-A column compartment H. The RP-HPLC separation was optimized based on the study conducted by Froning *et al.*[4] and performed on a Nucleodur Sphinx column (50 × 2.0 mm, 1.8 µm; Macherey-Nagel, Düren, Germany) with the solvents A: 30 mM $NH_4FA$ in MeOH/$H_2O$ (90:10, *v*/*v*) + 0.1% FA and B: 30 mM $NH_4FA$ in IPA/MeOH (95:5, *v*/*v*) + 0.1% FA for a 15 min gradient elution. The gradient started and maintained at 0 %B for 0.5 min, before increasing to 80 %B in 10 min. 80 %B was kept for 1.5 min as rinsing step. For equilibration, the solvents were changed back to 0 %B in 0.5 min and maintained at this level for 2.5 min. Additionally, the RP-HPLC included 500 µL/min flow rate, 2 µL injection volume, and 40 °C column temperature.

**LC-TIMS-MS/MS:** Data dependent acquisition (dda)-PASEF was utilized with 1.50 *m/z* isolation width and varying collision energies for each technical replicate (negative ionization mode: 80 eV, 90 eV, 100 eV; positive ionization mode: 40 eV, 60 eV, 80 eV). Furthermore, fragmentation selection was limited to 4000 target intensity, 100 intensity threshold, *m/z* 600 – 2300 mass range and 0-1 charge range. The active exclusion was set to 0.1 min, and the number of PASEF scans per cycle was set to 2, resulting in 1.52 s total cycle time.

## SI-4: Data processing

For data evaluation, the open-source MZmine 4.1 software[5] (mzio GmbH, Bremen, Germany) was utilized. Individual batch files were created for MALDI-TIMS-MS/MS and LC-TIMS-MS/MS in both positive and negative ionization mode, which were subsequently aligned to validate the results. Additionally, data evaluation was confirmed in DataAnalysis 6.1 (Bruker Daltonics GmbH & Co. KG, Bremen, Germany). The batch files are provided as mzbatch files and in written form, as described in the following.

**MALDI-TIMS-MS/MS data processing:** 5e1 (MS1), 0e0 (IMS), and 5e0 (MS2) noise level were set in negative ionization mode and 1e2 (MS1), 5e0 (IMS), and 1e1 (MS2) in positive ionization mode. MALDI spot feature detection was performed with 15 ppm mass tolerance and 1e2 minimum intensity. For mobilogram summing, the data were binned by factor 10 and subsequently smoothed by factor 5 Savitzky-Golay smoothing. Local minimum feature resolving in the mobility dimension was performed for a minimum of three data points with 70 % chromatographic threshold, 0.008 minimum search range, 1e1 minimum intensity, and 1.7 minimum peak ratio of top to edge. Mobilograms were further smoothed by factor 7 Savitzky-Golay smoothing. The $^{13}C$ isotope filter module and the isotopic peaks finder (H, C, N, O, S, P) were used to remove isotopes based on 3 ppm mass tolerance. Additionally, for the $^{13}C$ isotope filter 0.03 Vs/cm$^2$ mobility tolerance were set and the representative isotope was determined based on the lowest *m*/*z*. MS2 scans were grouped to their respective features based on 20 ppm mass tolerance and combined into a single spectrum. For joined feature alignment, 8 ppm mass tolerance (weight 3) and 0.01 Vs/cm$^2$ mobility tolerance (weight 1) were set. For further feature list cleaning, the feature list rows filter (keep rows with MS2), the (multithreaded) peak finder (gap filling with 20 % intensity tolerance and 20 ppm mass tolerance for a minimum of one data point), and the duplicate peak filter (new average mode, 1.5 ppm mass tolerance, 0.008 Vs/cm$^2$ mobility tolerance) were utilized. Lipid annotation was performed using custom lipid classes for lipid A (**Table S2**) containing C10 – C16 side chains and no double bonds with 8 ppm mass tolerance (MS1) and 20 ppm mass tolerance (MS2).

**LC-TIMS-MS/MS data processing:** 5e1 (MS1), 0e0 (IMS), and 5e0 (MS2) noise level were set. Chromatogram building was performed using 1e2 group intensity for a minimum of four consecutive scans, 1e2 minimum intensity, and 20 ppm mass tolerance.[6] Chromatograms were further smoothed by factor 5 Savitzky-Golay smoothing. Local minimum feature resolving in the retention time dimension was performed for a minimum of four data points with 90 % chromatographic threshold, 0.05 minimum search range, 1e2 minimum intensity, and 1.8 minimum peak ratio of top to edge. Mobilograms were added by the IMS expander using 20 ppm mass tolerance. Data were binned by factor 10 and subsequently smoothed by factor 5 Savitzky-Golay smoothing. Local minimum feature resolving in the mobility dimension was performed for a minimum of five data points with 70 % chromatographic threshold, 0.008 minimum search range, 1e1 minimum intensity, and 1.7 minimum peak ratio of top to edge. Mobilograms and chromatograms were further smoothed by factor 5 Savitzky-Golay smoothing. The $^{13}C$ isotope filter module and the isotopic peaks finder (H, C, N, O, S, P) were used to remove isotopes based on 3 ppm mass tolerance. Additionally, for the $^{13}C$ isotope filter 0.04 min retention time tolerance and 0.008 Vs/cm$^2$ mobility tolerance were set, features with MS2 spectra were not removed, and the representative isotope was determined based on the lowest *m*/*z*. For joined feature alignment, 8 ppm mass tolerance (weight 3), 0.1 min retention time tolerance (weight 1) and 0.01 Vs/cm$^2$ mobility tolerance (weight 1) were set. For further feature list cleaning, the feature list rows filter (keep rows with MS2), the (multithreaded) peak finder (gap filling with 20 % intensity tolerance, 20 ppm mass tolerance, and 0.1 min retention time tolerance for a minimum of one data point), and the duplicate peak filter (new average mode, 1.5 ppm mass tolerance, 0.035 min retention time tolerance, and 0.008 Vs/cm$^2$ mobility tolerance) were utilized. Lipid annotation was performed using custom lipid classes for lipid A (**Table S2**) containing C10 – C16 side chains and no double bonds with 8 ppm mass tolerance (MS1) and 20 ppm mass tolerance (MS2).

To validate the MALDI-TIMS-MS/MS data with the results of the LC-TIMS-MS/MS experiments, the LC image aligner module was used with 5 ppm mass tolerance (weight 1) and 0.008 Vs/cm$^2$ mobility tolerance (weight 1).

## Custom lipid A classes

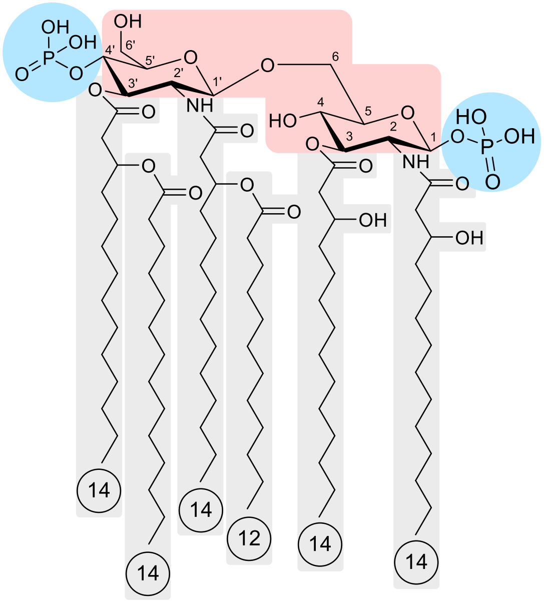


**Figure S1:** Structural formula of the major lipid A species in *E. coli*, also known as 6-acyl diphosphorylated lipid A. In general, lipid A species consist of a disaccharide backbone (red), polar head groups (blue), and a varying number of fatty acid residues (gray). The circled numbers below the side chains display the number of carbon atoms in the respective chain.

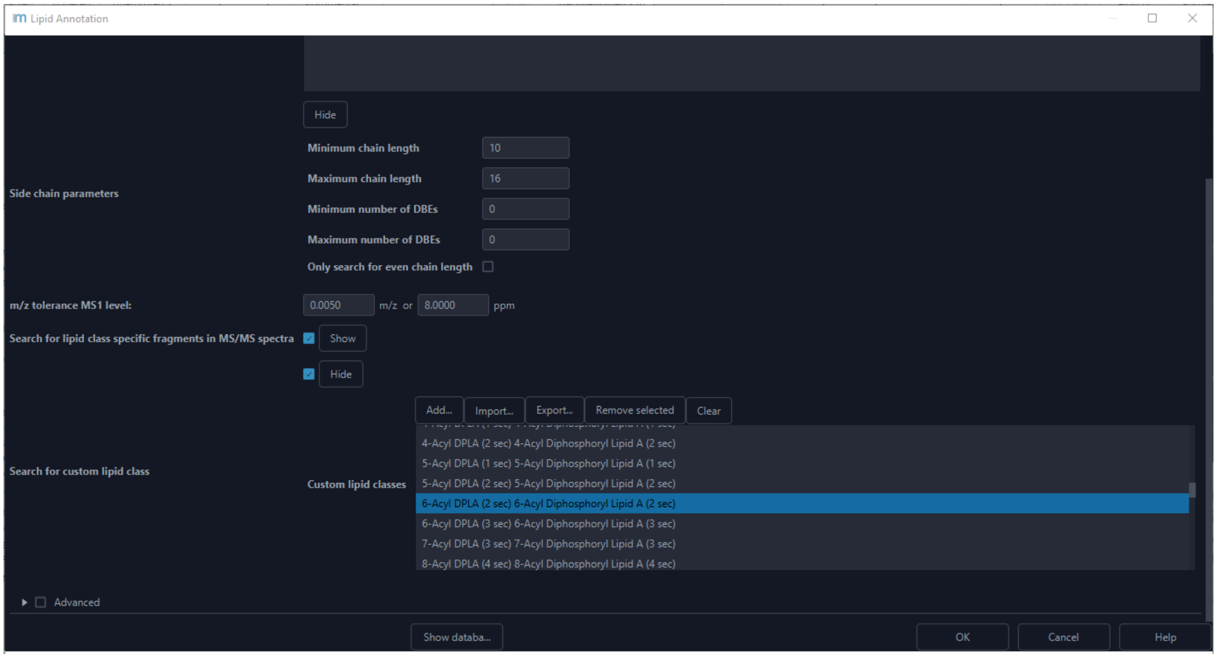


**Figure S2:** Overview of the lipid annotation surface in the mzmine software package highlighting the custom lipid class addition. In the lower bottom of this figure, an overview of the created custom lipid classes for annotation is summarized. Custom lipid classes can be added based on new or modified preset lipid classes. Furthermore, individual custom lipid classes or custom lipid class batches can be imported or exported as .json files for user-friendly sharing. Note that the developed custom lipid A classes of this study are shared publicly available on MassIVE MSV000096293 (https://doi.org/doi:10.25345/C5707X11F).

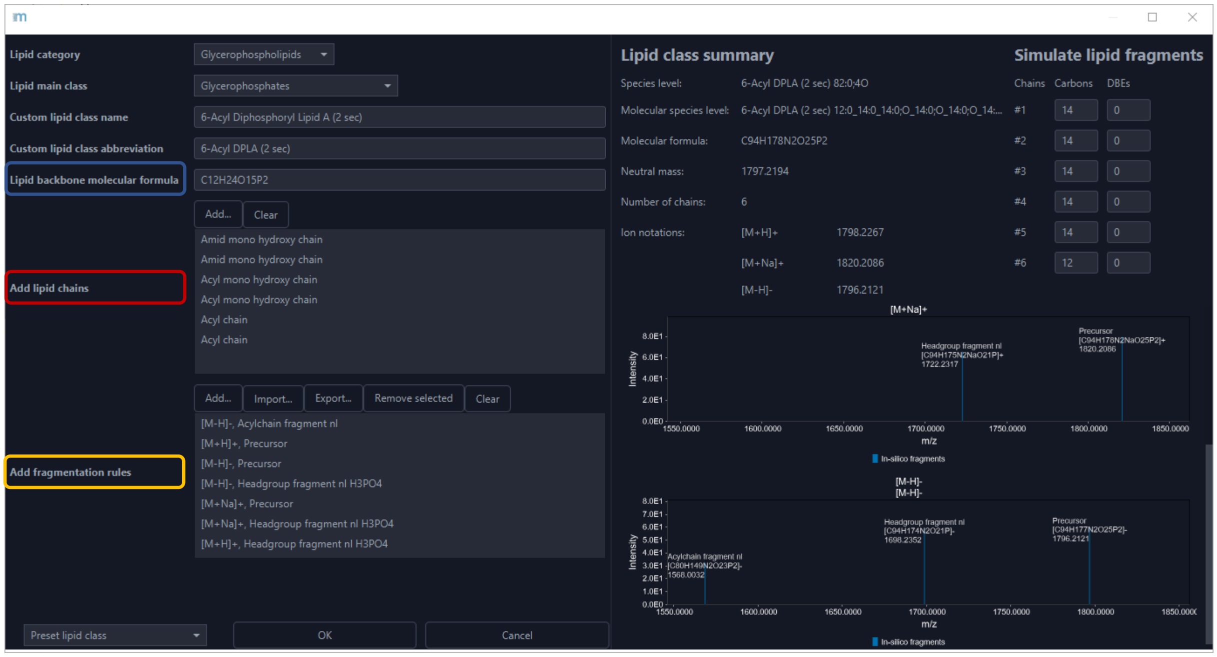


**Figure S3:** Overview of the custom lipid class surface in the mzmine software package exemplified for 6-acyl DPLA (2 sec). Left side (from top to bottom): The custom lipid class can be categorized and defined according to LIPID MAPS hierarchy. Furthermore, it can be named including an abbreviation. To define the custom lipid class, the backbone formula (highlighted in blue) and the side chain combinations including the type of chain (highlighted in red) are required. Moreover, fragmentation rules for user-defined adducts can be added (highlighted in orange). Right side (from top to bottom): The custom lipid class is summarized including one exemplified lipid species (here: the major lipid A species in *E.* coli) based on the predefined carbon atoms and double bonds. Moreover, simulated MS/MS spectra are illustrated of predefined adducts for verification.

**Table S2:** Overview of the 72 custom lipid A classes developed in mzmine for automated lipid A profiling including custom lipid class name, lipid backbone, number and type of side chains (amide mono hydroxy chains, acyl mono hydoxy chains, and acyl chains) as well as the fragmentation rules in negative and positive ionization modes (NL were used as neutral loss abbreviation).

| Custom lipid class | Lipid backbone | Amide mono hydroxy chain | Acyl mono hydroxy chain | Acyl chain | Fragmentation rules $[M-H]^-$ | Fragmentation rules $[M+H]^+$ and $[M+Na]^+$ |
|---|---|---|---|---|---|---|
| 2-acyl MPLA (0 sec) | $C_{12}H_{23}O_{12}P_1$ | 2x | - | - | precursor | precursor, NL $H_3PO_4$ headgroup (C1P) or NL $H_2O$ headgroup (C4'P) |
| 3-acyl MPLA (0 sec) | $C_{12}H_{23}O_{12}P_1$ | 2x | 1x | - | precursor, NL acylchain | precursor, NL $H_3PO_4$ headgroup (C1P) or NL $H_2O$ headgroup (C4'P) |
| 3-acyl MPLA (1 sec) | $C_{12}H_{23}O_{12}P_1$ | 2x | - | 1x | precursor, NL acylchain | precursor, NL $H_3PO_4$ headgroup (C1P) or NL $H_2O$ headgroup (C4'P) |
| 4-acyl MPLA (0 sec) | $C_{12}H_{23}O_{12}P_1$ | 2x | 2x | - | precursor, NL acylchain | precursor, NL $H_3PO_4$ headgroup (C1P) or NL $H_2O$ headgroup (C4'P) |
| 4-acyl MPLA (1 sec) | $C_{12}H_{23}O_{12}P_1$ | 2x | 1x | 1x | precursor, NL acylchain | precursor, NL $H_3PO_4$ headgroup (C1P) or NL $H_2O$ headgroup (C4'P) |
| 4-acyl MPLA (2 sec) | $C_{12}H_{23}O_{12}P_1$ | 2x | - | 2x | precursor, NL acylchain | precursor, NL $H_3PO_4$ headgroup (C1P) or NL $H_2O$ headgroup (C4'P) |
| 5-acyl MPLA (1 sec) | $C_{12}H_{23}O_{12}P_1$ | 2x | 2x | 1x | precursor, NL acylchain | precursor, NL $H_3PO_4$ headgroup (C1P) or NL $H_2O$ headgroup (C4'P) |
| 5-acyl MPLA (2 sec) | $C_{12}H_{23}O_{12}P_1$ | 2x | 1x | 2x | precursor, NL acylchain | precursor, NL $H_3PO_4$ headgroup (C1P) or NL $H_2O$ headgroup (C4'P) |
| 6-acyl MPLA (2 sec) | $C_{12}H_{23}O_{12}P_1$ | 2x | 2x | 2x | precursor, NL acylchain | precursor, NL $H_3PO_4$ headgroup (C1P) or NL $H_2O$ headgroup (C4'P) |
| 6-acyl MPLA (3 sec) | $C_{12}H_{23}O_{12}P_1$ | 2x | 1x | 3x | precursor, NL acylchain | precursor, NL $H_3PO_4$ headgroup (C1P) or NL $H_2O$ headgroup (C4'P) |
| 7-acyl MPLA (3 sec) | $C_{12}H_{23}O_{12}P_1$ | 2x | 2x | 3x | precursor, NL acylchain | precursor, NL $H_3PO_4$ headgroup (C1P) or NL $H_2O$ headgroup (C4'P) |
| 8-acyl MPLA (4 sec) | $C_{12}H_{23}O_{12}P_1$ | 2x | 2x | 4x | precursor, NL acylchain | precursor, NL $H_3PO_4$ headgroup (C1P) or NL $H_2O$ headgroup (C4'P) |
| 2-acyl DPLA (0 sec) | $C_{12}H_{24}O_{15}P_2$ | 2x | - | - | precursor, NL $H_3PO_4$ headgroup | precursor, NL $H_3PO_4$ headgroup |
| 3-acyl DPLA (0 sec) | $C_{12}H_{24}O_{15}P_2$ | 2x | 1x | - | precursor, NL acylchain, NL $H_3PO_4$ headgroup | precursor, NL $H_3PO_4$ headgroup |
| 3-acyl DPLA (1 sec) | $C_{12}H_{24}O_{15}P_2$ | 2x | - | 1x | precursor, NL acylchain, NL $H_3PO_4$ headgroup | precursor, NL $H_3PO_4$ headgroup |
| 4-acyl DPLA (0 sec) | $C_{12}H_{24}O_{15}P_2$ | 2x | 2x | - | precursor, NL acylchain, NL $H_3PO_4$ headgroup | precursor, NL $H_3PO_4$ headgroup |
| 4-acyl DPLA (1 sec) | $C_{12}H_{24}O_{15}P_2$ | 2x | 1x | 1x | precursor, NL acylchain, NL $H_3PO_4$ headgroup | precursor, NL $H_3PO_4$ headgroup |
| 4-acyl DPLA (2 sec) | $C_{12}H_{24}O_{15}P_2$ | 2x | - | 2x | precursor, NL acylchain, NL $H_3PO_4$ headgroup | precursor, NL $H_3PO_4$ headgroup |
| 5-acyl DPLA (1 sec) | $C_{12}H_{24}O_{15}P_2$ | 2x | 2x | 1x | precursor, NL acylchain, NL $H_3PO_4$ headgroup | precursor, NL $H_3PO_4$ headgroup |
| 5-acyl DPLA (2 sec) | $C_{12}H_{24}O_{15}P_2$ | 2x | 1x | 2x | precursor, NL acylchain, | precursor, NL $H_3PO_4$ headgroup |

| | | | | | | |
|---|---|---|---|---|---|---|
| | | | | | NL $H_3PO_4$ headgroup | |
| 6-acyl DPLA (2 sec) | $C_{12}H_{24}O_{15}P_2$ | 2x | 2x | 2x | precursor, NL acylchain, NL $H_3PO_4$ headgroup | precursor, NL $H_3PO_4$ headgroup |
| 6-acyl DPLA (3 sec) | $C_{12}H_{24}O_{15}P_2$ | 2x | 1x | 3x | precursor, NL acylchain, NL $H_3PO_4$ headgroup | precursor, NL $H_3PO_4$ headgroup |
| 7-acyl DPLA (3 sec) | $C_{12}H_{24}O_{15}P_2$ | 2x | 2x | 3x | precursor, NL acylchain, NL $H_3PO_4$ headgroup | precursor, NL $H_3PO_4$ headgroup |
| 8-acyl DPLA (4 sec) | $C_{12}H_{24}O_{15}P_2$ | 2x | 2x | 4x | precursor, NL acylchain, NL $H_3PO_4$ headgroup | precursor, NL $H_3PO_4$ headgroup |
| 2-acyl MPLA (0 sec) PEtN | $C_{14}H_{29}N_1O_{15}P_2$ | 2x | - | - | precursor, NL $C_2H_6N_1O_3P_1$ headgroup | precursor |
| 3-acyl MPLA (0 sec) PEtN | $C_{14}H_{29}N_1O_{15}P_2$ | 2x | 1x | - | precursor, NL $C_2H_6N_1O_3P_1$ headgroup | precursor |
| 3-acyl MPLA (1 sec) PEtN | $C_{14}H_{29}N_1O_{15}P_2$ | 2x | - | 1x | precursor, NL $C_2H_6N_1O_3P_1$ headgroup | precursor |
| 4-acyl MPLA (0 sec) PEtN | $C_{14}H_{29}N_1O_{15}P_2$ | 2x | 2x | - | precursor, NL $C_2H_6N_1O_3P_1$ headgroup | precursor |
| 4-acyl MPLA (1 sec) PEtN | $C_{14}H_{29}N_1O_{15}P_2$ | 2x | 1x | 1x | precursor, NL $C_2H_6N_1O_3P_1$ headgroup | precursor |
| 4-acyl MPLA (2 sec) PEtN | $C_{14}H_{29}N_1O_{15}P_2$ | 2x | - | 2x | precursor, NL $C_2H_6N_1O_3P_1$ headgroup | precursor |
| 5-acyl MPLA (1 sec) PEtN | $C_{14}H_{29}N_1O_{15}P_2$ | 2x | 2x | 1x | precursor, NL $C_2H_6N_1O_3P_1$ headgroup | precursor |
| 5-acyl MPLA (2 sec) PEtN | $C_{14}H_{29}N_1O_{15}P_2$ | 2x | 1x | 2x | precursor, NL $C_2H_6N_1O_3P_1$ headgroup | precursor |
| 6-acyl MPLA (2 sec) PEtN | $C_{14}H_{29}N_1O_{15}P_2$ | 2x | 2x | 2x | precursor, NL $C_2H_6N_1O_3P_1$ headgroup | precursor |
| 6-acyl MPLA (3 sec) PEtN | $C_{14}H_{29}N_1O_{15}P_2$ | 2x | 1x | 3x | precursor, NL $C_2H_6N_1O_3P_1$ headgroup | precursor |
| 7-acyl MPLA (3 sec) PEtN | $C_{14}H_{29}N_1O_{15}P_2$ | 2x | 2x | 3x | precursor, NL $C_2H_6N_1O_3P_1$ headgroup | precursor |
| 8-acyl MPLA (4 sec) PEtN | $C_{14}H_{29}N_1O_{15}P_2$ | 2x | 2x | 4x | precursor, NL $C_2H_6N_1O_3P_1$ headgroup | precursor |
| 2-acyl DPLA (0 sec) PEtN | $C_{14}H_{30}N_1O_{18}P_3$ | 2x | - | - | precursor, NL $C_2H_6N_1O_3P_1$ headgroup, NL $H_3PO_4$ headgroup | precursor |
| 3-acyl DPLA (0 sec) PEtN | $C_{14}H_{30}N_1O_{18}P_3$ | 2x | 1x | - | precursor, NL $C_2H_6N_1O_3P_1$ headgroup, NL $H_3PO_4$ headgroup | precursor |
| 3-acyl DPLA (1 sec) PEtN | $C_{14}H_{30}N_1O_{18}P_3$ | 2x | - | 1x | precursor, NL $C_2H_6N_1O_3P_1$ headgroup, NL $H_3PO_4$ headgroup | precursor |
| 4-acyl DPLA (0 sec) PEtN | $C_{14}H_{30}N_1O_{18}P_3$ | 2x | 2x | - | precursor, NL $C_2H_6N_1O_3P_1$ headgroup, NL $H_3PO_4$ headgroup | precursor |
| 4-acyl DPLA (1 sec) PEtN | $C_{14}H_{30}N_1O_{18}P_3$ | 2x | 1x | 1x | precursor, NL $C_2H_6N_1O_3P_1$ headgroup, NL $H_3PO_4$ headgroup | precursor |
| 4-acyl DPLA (2 sec) PEtN | $C_{14}H_{30}N_1O_{18}P_3$ | 2x | - | 2x | precursor, NL $C_2H_6N_1O_3P_1$ headgroup, NL $H_3PO_4$ headgroup | precursor |
| 5-acyl DPLA (1 sec) PEtN | $C_{14}H_{30}N_1O_{18}P_3$ | 2x | 2x | 1x | precursor, NL $C_2H_6N_1O_3P_1$ headgroup, NL $H_3PO_4$ headgroup | precursor |

| | | | | | | |
|---|---|---|---|---|---|---|
| 5-acyl DPLA (2 sec) PEtN | $C_{14}H_{30}N_1O_{18}P_3$ | 2x | 1x | 2x | precursor, NL $C_2H_6N_1O_3P_1$ headgroup, NL $H_3PO_4$ headgroup | precursor |
| 6-acyl DPLA (2 sec) PEtN | $C_{14}H_{30}N_1O_{18}P_3$ | 2x | 2x | 2x | precursor, NL $C_2H_6N_1O_3P_1$ headgroup, NL $H_3PO_4$ headgroup | precursor |
| 6-acyl DPLA (3 sec) PEtN | $C_{14}H_{30}N_1O_{18}P_3$ | 2x | 1x | 3x | precursor, NL $C_2H_6N_1O_3P_1$ headgroup, NL $H_3PO_4$ headgroup | precursor |
| 7-acyl DPLA (3 sec) PEtN | $C_{14}H_{30}N_1O_{18}P_3$ | 2x | 2x | 3x | precursor, NL $C_2H_6N_1O_3P_1$ headgroup, NL $H_3PO_4$ headgroup | precursor |
| 8-acyl DPLA (4 sec) PEtN | $C_{14}H_{30}N_1O_{18}P_3$ | 2x | 2x | 4x | precursor, NL $C_2H_6N_1O_3P_1$ headgroup, NL $H_3PO_4$ headgroup | precursor |
| 2-acyl MPLA (0 sec) AraN | $C_{17}H_{32}N_1O_{15}P_1$ | 2x | - | - | precursor, NL $C_5H_9N_1O_3$ headgroup | precursor |
| 3-acyl MPLA (0 sec) AraN | $C_{17}H_{32}N_1O_{15}P_1$ | 2x | 1x | - | precursor, NL $C_5H_9N_1O_3$ headgroup | precursor |
| 3-acyl MPLA (1 sec) AraN | $C_{17}H_{32}N_1O_{15}P_1$ | 2x | - | 1x | precursor, NL $C_5H_9N_1O_3$ headgroup | precursor |
| 4-acyl MPLA (0 sec) AraN | $C_{17}H_{32}N_1O_{15}P_1$ | 2x | 2x | - | precursor, NL $C_5H_9N_1O_3$ headgroup | precursor |
| 4-acyl MPLA (1 sec) AraN | $C_{17}H_{32}N_1O_{15}P_1$ | 2x | 1x | 1x | precursor, NL $C_5H_9N_1O_3$ headgroup | precursor |
| 4-acyl MPLA (2 sec) AraN | $C_{17}H_{32}N_1O_{15}P_1$ | 2x | - | 2x | precursor, NL $C_5H_9N_1O_3$ headgroup | precursor |
| 5-acyl MPLA (1 sec) AraN | $C_{17}H_{32}N_1O_{15}P_1$ | 2x | 2x | 1x | precursor, NL $C_5H_9N_1O_3$ headgroup | precursor |
| 5-acyl MPLA (2 sec) AraN | $C_{17}H_{32}N_1O_{15}P_1$ | 2x | 1x | 2x | precursor, NL $C_5H_9N_1O_3$ headgroup | precursor |
| 6-acyl MPLA (2 sec) AraN | $C_{17}H_{32}N_1O_{15}P_1$ | 2x | 2x | 2x | precursor, NL $C_5H_9N_1O_3$ headgroup | precursor |
| 6-acyl MPLA (3 sec) AraN | $C_{17}H_{32}N_1O_{15}P_1$ | 2x | 1x | 3x | precursor, NL $C_5H_9N_1O_3$ headgroup | precursor |
| 7-acyl MPLA (3 sec) AraN | $C_{17}H_{32}N_1O_{15}P_1$ | 2x | 2x | 3x | precursor, NL $C_5H_9N_1O_3$ headgroup | precursor |
| 8-acyl MPLA (4 sec) AraN | $C_{17}H_{32}N_1O_{15}P_1$ | 2x | 2x | 4x | precursor, NL $C_5H_9N_1O_3$ headgroup | precursor |
| 2-acyl DPLA (0 sec) AraN | $C_{17}H_{33}N_1O_{18}P_2$ | 2x | - | - | precursor, NL $C_5H_9N_1O_3$ headgroup, NL $H_3PO_4$ headgroup | precursor |
| 3-acyl DPLA (0 sec) AraN | $C_{17}H_{33}N_1O_{18}P_2$ | 2x | 1x | - | precursor, NL $C_5H_9N_1O_3$ headgroup, NL $H_3PO_4$ headgroup | precursor |
| 3-acyl DPLA (1 sec) AraN | $C_{17}H_{33}N_1O_{18}P_2$ | 2x | - | 1x | precursor, NL $C_5H_9N_1O_3$ headgroup, NL $H_3PO_4$ headgroup | precursor |
| 4-acyl DPLA (0 sec) AraN | $C_{17}H_{33}N_1O_{18}P_2$ | 2x | 2x | - | precursor, NL $C_5H_9N_1O_3$ headgroup, NL $H_3PO_4$ headgroup | precursor |
| 4-acyl DPLA (1 sec) AraN | $C_{17}H_{33}N_1O_{18}P_2$ | 2x | 1x | 1x | precursor, NL $C_5H_9N_1O_3$ headgroup, NL $H_3PO_4$ headgroup | precursor |
| 4-acyl DPLA (2 sec) AraN | $C_{17}H_{33}N_1O_{18}P_2$ | 2x | - | 2x | precursor, NL $C_5H_9N_1O_3$ headgroup, NL $H_3PO_4$ headgroup | precursor |
| 5-acyl DPLA (1 sec) AraN | $C_{17}H_{33}N_1O_{18}P_2$ | 2x | 2x | 1x | precursor, NL $C_5H_9N_1O_3$ headgroup, NL $H_3PO_4$ headgroup | precursor |

| | | | | | | |
|---|---|---|---|---|---|---|
| 5-acyl DPLA (2 sec) AraN | $C_{17}H_{33}N_1O_{18}P_2$ | 2x | 1x | 2x | precursor, NL $C_5H_9N_1O_3$ headgroup, NL $H_3PO_4$ headgroup | precursor |
| 6-acyl DPLA (2 sec) AraN | $C_{17}H_{33}N_1O_{18}P_2$ | 2x | 2x | 2x | precursor, NL $C_5H_9N_1O_3$ headgroup, NL $H_3PO_4$ headgroup | precursor |
| 6-acyl DPLA (3 sec) AraN | $C_{17}H_{33}N_1O_{18}P_2$ | 2x | 1x | 3x | precursor, NL $C_5H_9N_1O_3$ headgroup, NL $H_3PO_4$ headgroup | precursor |
| 7-acyl DPLA (3 sec) AraN | $C_{17}H_{33}N_1O_{18}P_2$ | 2x | 2x | 3x | precursor, NL $C_5H_9N_1O_3$ headgroup, NL $H_3PO_4$ headgroup | precursor |
| 8-acyl DPLA (4 sec) AraN | $C_{17}H_{33}N_1O_{18}P_2$ | 2x | 2x | 4x | precursor, NL $C_5H_9N_1O_3$ headgroup, NL $H_3PO_4$ headgroup | precursor |

## SI-5: MALDI-TIMS-MS/MS method development

The DPLA standard extract from *E. coli* F583 was employed to test different MALDI matrices. For this purpose, the MALDI matrices sinapic acid (SA, ≥ 99.0 %), α-cyano-4-hydroxycinnamic acid (CHCA, 97 %), *trans*-ferulic acid (FA, 99 %), 4-chloro-α-cyanocinnamic acid (CCA, ≥ 95.0 %), 2,5-dihydroxybenzoic acid (DHB, 97 %), 2,6-dihydroxyacetophenone (DHAP, 97 %), and 9-aminoacridine (9-AA, ≥ 99.5 %) were provided by Sigma-Aldrich (Steinheim, Germany), and 6-aza-2-thiothymine (ATT, 98 %) from Thermo Fisher Scientific (Kandel, Germany). The low purity of the DPLA standard extract provides a distinct advantage in method development, as it allows for evaluation of multiple lipid A species. The matrix evaluation encompassed the sensitivity for lipid A analysis, the number of interfering isobars resulting from the matrix application, and the homogeneity of the matrix crystal distribution for MALDI spot analysis. The samples were spotted on the MALDI target plate according to the "sandwich" procedure (i.e., the sample is spotted on the MALDI target, and then embedded by the MALDI matrix), and subsequently analyzed using dried droplet MALDI. In negative ionization mode, the three matrices 9-AA, DHAP, and DHB were tested. The results obtained for 9-AA were the most favorable, exhibiting remarkably high sensitivity for the DPLA species, a small number of interfering ions, and high degree of homogeneity (**Figure S4a and S5b**). In positive ionization mode, seven matrices were evaluated, including ATT, CCA, CHCA, DHAP, DHB, FA, and SA. In this ionization mode, the most favorable outcomes were observed using FA as MALDI matrix (**Figure S4b and S5a**). However, the sensitivity is considerably diminished compared to the negative ionization mode and the number of interfering ions caused by the matrix application is significantly higher. Nevertheless, the positive ionization mode may provide additional insights for lipid A characterization, and therefore, its implementation in MALDI spot analysis is of particular relevance as a complementary method to negative ionization mode MALDI.

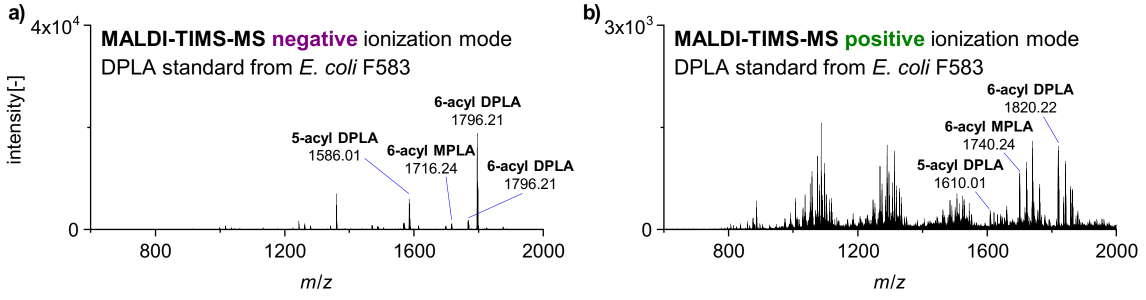


**Figure S4:** MALDI-TIMS-MS spectra of the DPLA standard extract from *E. coli* F583 using a) 9-AA as matrix in negative ionization mode and b) FA as matrix in positive ionization mode.

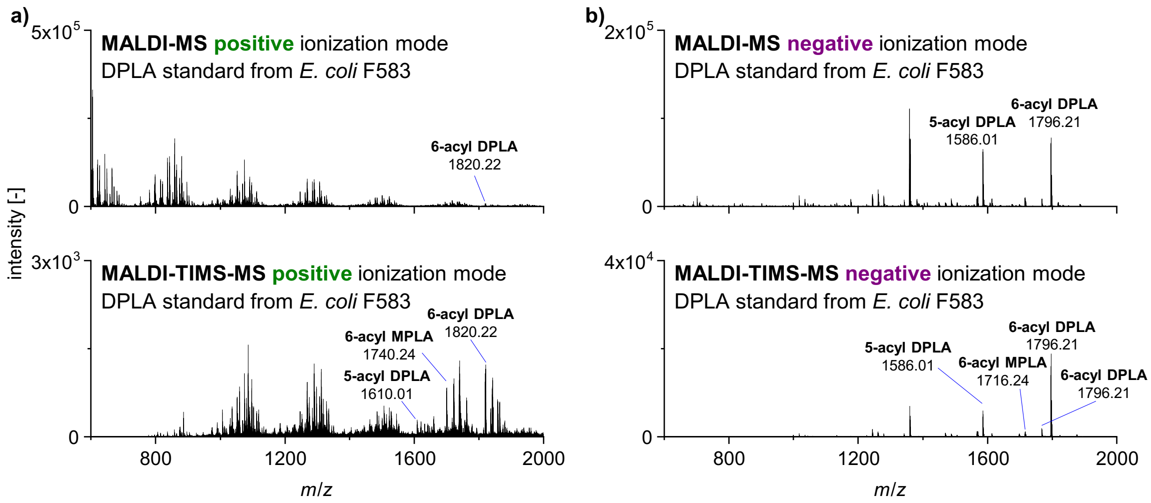


**Figure S5:** MALDI-MS (top) and MALDI-TIMS-MS (bottom) spectra of the DPLA standard extract from *E. coli* F583 using a) FA as matrix in positive ionization mode and b) 9-AA as matrix in negative ionization mode. Using TIMS, a sensitivity enhancement for lipid A analysis was provided.

In addition to the matrix evaluation, the microextraction was optimized regarding the hydrolysis time and the incorporation of an additional extraction step, aiming to improve the sensitivity. For this purpose, a microextraction of *E. coli* CFT073 was performed under a variety of experimental conditions. To isolate lipid A from its oligosaccharide core, a mild acid hydrolysis is conducted using a citrate buffer. During method development, a better sensitivity was revealed for the citrate extraction in comparison to the more commonly utilized acetate/SDS buffer extraction[1]. Furthermore, the influence of the hydrolysis time on the hydrolysis yield was examined (**Figure S6a**). As a compromise between hydrolysis yield and time efficiency, the best results were attributed to a hydrolysis time of 60 min.

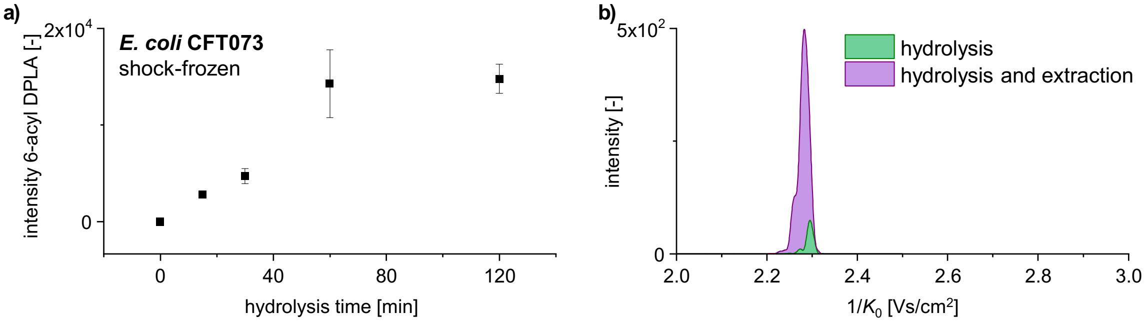


**Figure S6:** Method optimization steps to develop an efficient microextraction prior to MALDI-TIMS-MS/MS analysis. a) Influence of the hydrolysis time on the hydrolysis yield and b) impact of an additional extraction step on the methods sensitivity for the major 6-acyl DPLA in shock-frozen *E. coli* CFT073 using MALDI-TIMS-MS in negative ionization mode. For the hydrolysis study, three biological replicates for each time point were measured as three technical replicates each.

Furthermore, the influence of an additional extraction step on the sensitivity of this method was investigated (**Figure S6b**). The extraction step was incorporated directly after the hydrolysis to remove oligosaccharides and other polar compounds and enrich the nonpolar lipid A for MALDI spot analysis. The lipid A extraction phase did not require transferal or evaporation, as it could be spotted directly with a laboratory pipette for sandwich-based MALDI spot analysis. A mixture of 400 µL $CHCl_3$/MeOH (1:1, *v*/*v*) was added followed by a 5 min centrifugation step for phase separation. The additional extraction step resulted in a significant enhancement in sensitivity. Moreover, the extraction benefits from the solubility of lipid A in the organic phase, which improves both the drying time and the homogeneity of the spot. For these reasons, the additional extraction step was further incorporated in the microextraction for MALDI spot analysis. To sum up, a MALDI approach for both negative and positive ionization modes was developed, including a rapid, tube-based microextraction. This microextraction saves several extraction steps compared to the extensive extraction for LC-MS/MS-based lipid A profiling (**Figure S7).**

In more detail, the LPS extraction to remove the lipids from the evaporated bacterial pellet was omitted, and the lipid A extraction to remove the oligosaccharides was reduced to one cycle in conjunction with the direct spotting of the lipid A extract on the MALDI target plate. Therefore, the microextraction resulted in an efficient and sensitive method for comprehensive MALDI-TIMS-MS/MS-based lipid A screening of bacterial samples.

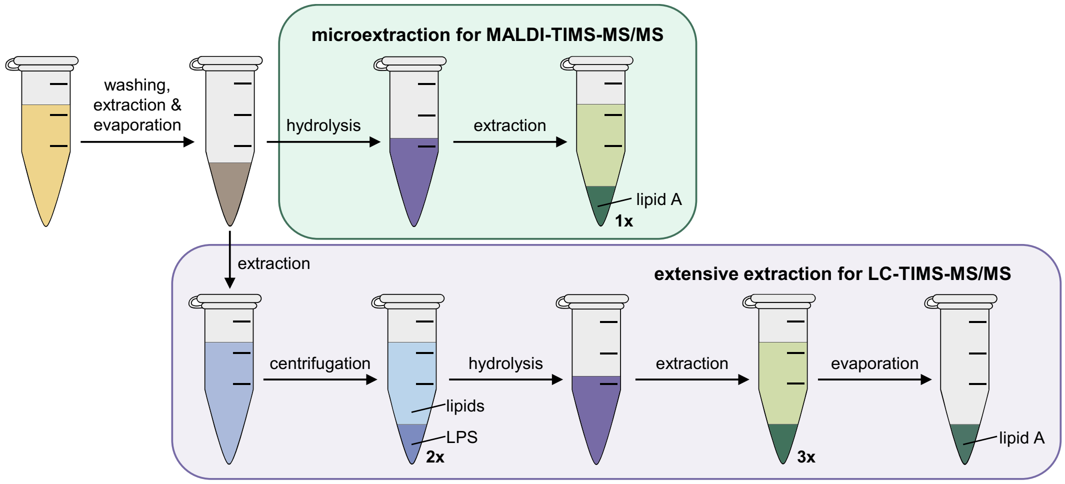


**Figure S7:** Sample preparation for the isolation of lipid A from bacterial strains using several washing and extraction steps as well as a mild acid hydrolysis. The developed efficient microextraction for MALDI-TIMS-MS/MS analysis is further compared with the extensive and more time-consuming extraction for LC-TIMS-MS/MS analysis.

A common challenge encountered in method transfer is that adduct formation differs between ESI- and MALDI-MS, particularly in positive ionization mode. Accordingly, the mobility separation for $[M+H]^+$ adducts of 6-acyl MPLA regioisomers were compared with that of $[M+NH_4]^+$ and $[M+Na]^+$ adducts (**Figure S8**). Notably, analogous differences in CCS of these regioisomers were observed for the $[M+Na]^+$ adducts ($\Delta$CCS = 2.2 %), whereas the $[M+NH_4]^+$ adducts provided similar mobility values for both regioisomers ($\Delta$CCS = 0.0 %). Furthermore, the MPLA C1P isomer was discriminated by the $[M+NH_4]^+$ adduct formation, whereas the $[M+Na]^+$ adducts of MPLA C4'P demonstrated a slight increase in response compared to the $[M+H]^+$ adducts. The successful separation of the $[M+Na]^+$ adducts is of particular interest, as sodiated lipid A adducts are commonly produced in MALDI spot analysis of bacterial samples. To confirm the results and examine the fragmentation pattern of sodiated MPLA adducts, MALDI-TIMS-MS/MS analysis of the DPLA standard extract was conducted. Once more, the $[M+Na]^+$ adducts of regioisomeric MPLA were resolved by TIMS, and specific fragments for regioisomeric MPLA with a mass shift of $\Delta m/z$ 80 were obtained by the cleavage of the C1 and C1' glycosidic bonds using mobility-resolved prm-PASEF. This demonstrates that MALDI-TIMS-MS/MS is an effective method for the direct characterization of regioisomeric MPLA in complex bacterial samples.

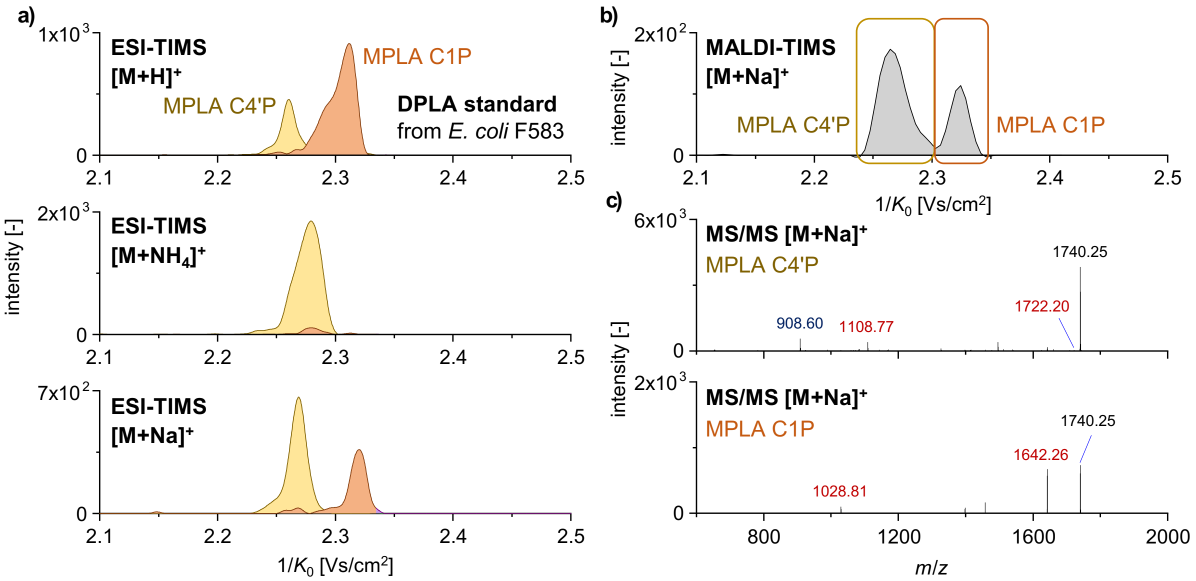


**Figure S8:** a) Influence of adduct formation on the TIMS separation of regioisomeric 6-acyl MPLA C4'P and MPLA C1P in the DPLA standard extract from *E. coli* F583 using LC-TIMS-MS/MS in positive ionization mode. b) MALDI-TIMS-MS/MS mobilogram of the sodiated regioisomers, and c) respective fragmentation spectra (60 eV, 70 eV, 80 eV combined).

## SI-6: LC-TIMS-MS/MS method development

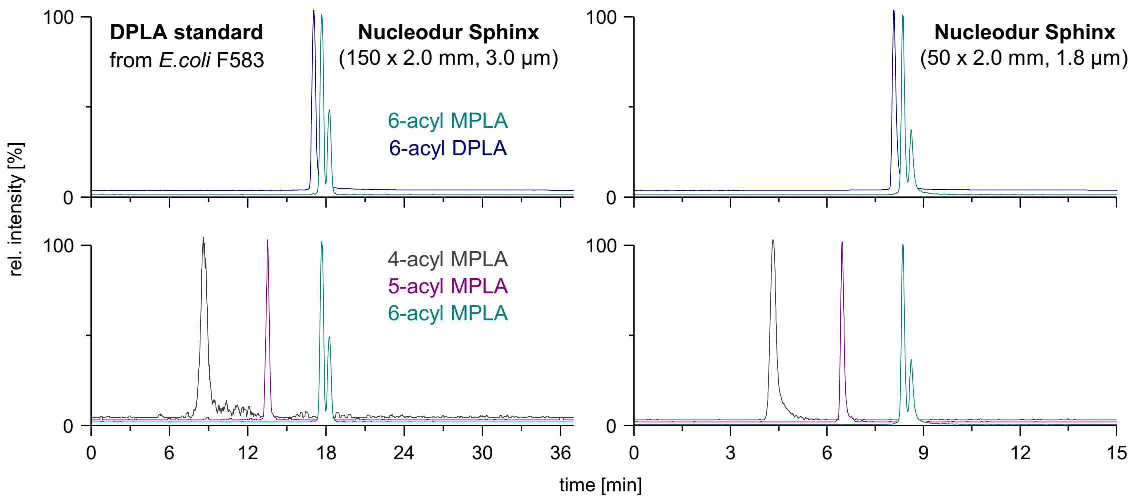


**Figure S9:** Transfer of the LC separation to a smaller Nucleodur Sphinx column for accelerated lipid A analysis with tailored separation of regioisomeric MPLA. Left: Separation of lipid A species depending on the phosphorylation number (top) and the acylation degree (bottom) on a 150 mm long Nucleodur Sphinx column with 3.0 µm particles and right: on a 50 mm short Nucleodur Sphinx column with 1.8 µm particles using the DPLA standard from *E. coli* F583. Note that the retention time scale differs depending on the used Nucleodur Sphinx column.

## SI-7: Comparison of identified lipid A species

Existing MALDI-based lipid A literature contains a limited number of structurally elucidated lipid A species, and in some cases, lacks CID experiments entirely.[7,8] Consequently, the observed identifications were compared to other literature pertaining to LC-MS/MS (see **Figure S10**).[8–11,4] In comparison to the existing literature, a substantial number of lipid A species were annotated through the implementation of our rapid screening method. While the majority of the literature employed acquisition in negative ionization mode, Sandor *et al.*[11] used a complementary approach based on both ionization modes. Therefore, a more detailed comparison of the IDs in relation to Sandor *et al.*[11] is illustrated in **Figure S11** below. Our LC-TIMS-MS/MS approach demonstrates comparable results in MPLA profiling to the LC-MS/MS approach from Sandor *et al.*[11]; however, the MALDI-TIMS-MS/MS approach exhibits enhanced performance in terms of structural elucidation of DPLA species. This advantage can be attributed to the challenging LC separation of diphosphorylated compounds, leading to tailing, and consequently, diminished sensitivity. In summary, our method demonstrated comparable results to the existing literature regarding the type and number of lipid A species, while offering benefits in the structural elucidation of DPLA species.

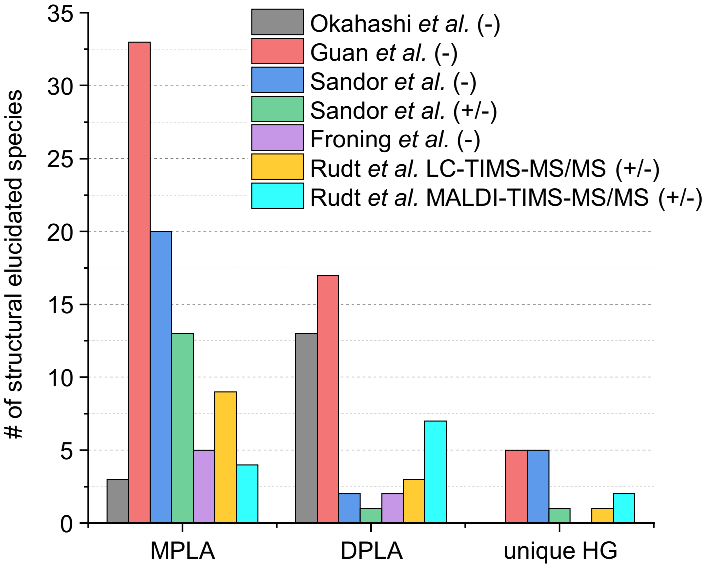


**Figure S10:** Comparison of the number of structural elucidated lipid A species in *E. coli* strains based on Okashashi *et al.*[9], Guan *et al.*[8], Sandor *et al.*[10], Sandor *et al.* (both ionization modes)[11], Froning *et al.*[4] as well as the LC-TIMS-MS/MS and MALDI-TIMS-MS/MS approach in this work.

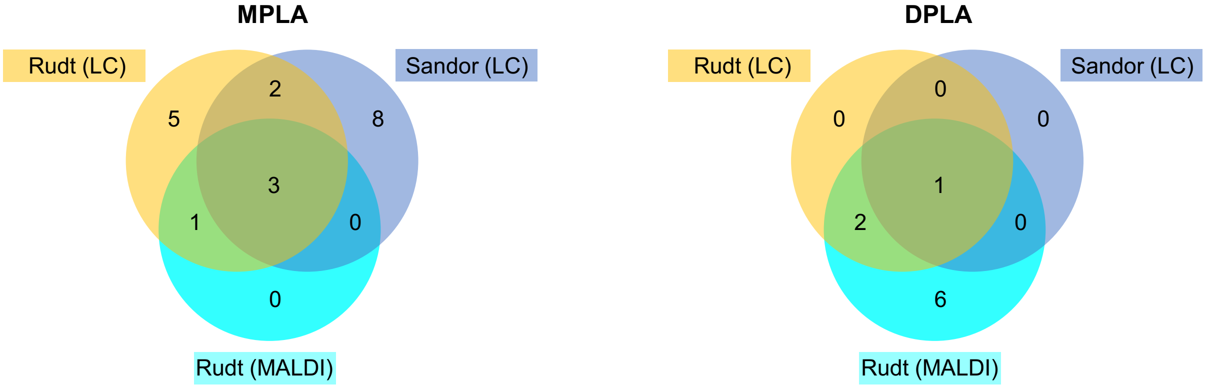


**Figure S11:** Detailed comparison of the number of structural elucidated MPLA and DPLA species in *E. coli* strains based on Sandor *et al.* (both ionization modes)[11] as well as the LC-TIMS-MS/MS and MALDI-TIMS-MS/MS approach in this work.

## SI-8: Identified lipid A species by MALDI-TIMS-MS/MS

**Table S3:** Elucidated lipid A structures by MALDI-TIMS-MS/MS in negative and positive ionization modes including accurate mass and headgroups or side chains attached to the disaccharide backbone. Lipid A species annotation was based on accurate mass, complementary fragmentation pattern in both ionization modes, and isotopic distribution. CCS values were utilized to confirm phosphorylation site in MPLA.

| Lipid A species | *m/z* [M-H]$^-$ | *m/z* [M+Na]$^+$ | C4' / C1 | C3' | C2' | C3 | C2 |
|---|---|---|---|---|---|---|---|
| 7-acyl DPLA (3 sec) 98:0;4O | 2034.4401 | 2058.4283 | 2x P | C14:0 (3-O-C14:0) | C14:0 (3-O-C12:0) | C14:0 (3-OH) | C14:0 (3-O-C16:0) |
| 6-acyl DPLA (2 sec) AraN 82:0;4O | 1927.2634 | | 2x P 1x AraN | C14:0 (3-O-C14:0) | C14:0 (3-O-C12:0) | C14:0 (3-OH) | C14:0 (3-OH) |
| 6-acyl DPLA (2 sec) PEtN 82:0;4O | 1919.2173 | | 2x P 1x PEtN | C14:0 (3-O-C14:0) | C14:0 (3-O-C12:0) | C14:0 (3-OH) | C14:0 (3-OH) |
| 6-acyl DPLA (2 sec) 84:0;4O | 1824.2391 | 1848.2315 | 2x P | C14:0 (3-O-C14:0) | C14:0 (3-O-C14:0) | C14:0 (3-OH) | C14:0 (3-OH) |
| 6-acyl DPLA (2 sec) 82:0;4O | 1796.2074 | 1820.1980 | 2x P | C14:0 (3-O-C14:0) | C14:0 (3-O-C12:0) | C14:0 (3-OH) | C14:0 (3-OH) |
| 6-acyl DPLA (2 sec) 80:0;4O | 1768.1758 | | 2x P | C14:0 (3-O-C12:0) | C14:0 (3-O-C12:0) | C14:0 (3-OH) | C14:0 (3-OH) |
| 6-acyl MPLA (2 sec) 84:0;4O | | 1768.2671 | C4'P | C14:0 (3-O-C14:0) | C14:0 (3-O-C14:0) | C14:0 (3-OH) | C14:0 (3-OH) |
| 6-acyl MPLA (2 sec) 82:0;4O | 1716.2406 | 1740.2358 | C1P | C14:0 (3-O-C14:0) | C14:0 (3-O-C12:0) | C14:0 (3-OH) | C14:0 (3-OH) |
| 6-acyl MPLA (2 sec) 82:0;4O | 1716.2406 | 1740.2331 | C4'P | C14:0 (3-O-C14:0) | C14:0 (3-O-C12:0) | C14:0 (3-OH) | C14:0 (3-OH) |
| 5-acyl DPLA (2 sec) 70:0;3O | 1598.0474 | | 2x P | C14:0 (3-O-C14:0) | C14:0 (3-O-C14:0) | | C14:0 (3-OH) |
| 5-acyl DPLA (2 sec) 68:0;3O | 1570.0125 | | 2x P | C14:0 (3-O-C14:0) | C14:0 (3-O-C12:0) | | C14:0 (3-OH) |
| 4-acyl DPLA (1 sec) 56:0;3O | 1387.8492 | | 2x P | | C14:0 (3-O-C14:0) | C14:0 (3-OH) | C14:0 (3-OH) |
| 4-acyl DPLA (1 sec) 54:0;3O | 1359.8162 | | 2x P | | C14:0 (3-O-C12:0) | C14:0 (3-OH) | C14:0 (3-OH) |
| 4-acyl MPLA (1 sec) 56:0;3O | 1307.8837 | 1331.8804 | C1P | | C14:0 (3-O-C14:0) | C14:0 (3-OH) | C14:0 (3-OH) |
| 4-acyl MPLA (1 sec) 54:0;3O | 1279.8499 | 1303.8442 | C1P | | C14:0 (3-O-C12:0) | C14:0 (3-OH) | C14:0 (3-OH) |
| 3-acyl DPLA (1 sec) 42:0;2O | 1161.6571 | | 2x P | | C14:0 (3-O-C14:0) | | C14:0 (3-OH) |
| 3-acyl DPLA (1 sec) 40:0;2O | 1133.6229 | 1157.6187 | 2x P | | C14:0 (3-O-C12:0) | | C14:0 (3-OH) |
| 3-acyl MPLA (1 sec) 42:0;2O | 1081.6892 | 1105.6833 | C1P | | C14:0 (3-O-C14:0) | | C14:0 (3-OH) |
| 3-acyl MPLA (1 sec) 40:0;2O | 1053.6560 | 1077.6506 | C1P | | C14:0 (3-O-C12:0) | | C14:0 (3-OH) |

**Table S4:** Identified lipid A species by MALDI-TIMS-MS/MS in negative ionization mode including accurate mass, mobility, CCS, explained MS2 pattern via custom lipid classes in mzmine, and mobility height for autoclaved and shock-frozen *E.* coli CFT073. Lipid A species annotation was based on accurate mass, fragmentation, and isotopic distribution. Species were identified as $[M-H]^-$ adducts.

| Lipid A species | *m*/*z* exp | $1/K_0$ [$Vs/cm^2$] | $CCS^{TIMS}$ [$Å^2$] | Explained MS2 intensity | height autoclaved [-] | height shock-frozen [-] |
|---|---|---|---|---|---|---|
| 7-acyl DPLA (3 sec) 98:0;4O | 2034.4401 | 2.470 | 498.0 | 100.0% | n.d. | 3.15E+02 |
| 6-acyl DPLA (2 sec) AraN 82:0;4O | 1927.2634 | 2.376 | 479.5 | 47.6% | n.d. | 7.72E+02 |
| 6-acyl DPLA (2 sec) PEtN 82:0;4O | 1919.2173 | 2.329 | 469.5 | 58.0% | n.d. | 4.00E+02 |
| 6-acyl DPLA (2 sec) 84:0;4O | 1824.2391 | 2.317 | 467.3 | 45.1% | 3.30E+01 | 1.15E+03 |
| 6-acyl DPLA (2 sec) 82:0;4O | 1796.2074 | 2.291 | 462.3 | 100.0% | 3.11E+02 | 5.13E+03 |
| 6-acyl DPLA (2 sec) 80:0;4O | 1768.1758 | 2.266 | 457.0 | 49.7% | n.d. | 5.46E+02 |
| 6-acyl MPLA (2 sec) 82:0;4O | 1716.2406 | 2.250 | 454.7 | 39.7% | n.d. | 5.15E+02 |
| 5-acyl DPLA (2 sec) 70:0;3O | 1598.0474 | 2.123 | 427.5 | 54.5% | 1.13E+02 | 2.20E+02 |
| 5-acyl DPLA (2 sec) 68:0;3O | 1570.0125 | 2.089 | 422.2 | 43.7% | 4.39E+02 | 8.67E+02 |
| 4-acyl DPLA (1 sec) 56:0;3O | 1387.8492 | 1.937 | 391.7 | 33.3% | 3.23E+02 | n.d. |
| 4-acyl DPLA (1 sec) 54:0;3O | 1359.8162 | 1.907 | 385.7 | 100.0% | 1.30E+03 | 3.55E+02 |
| 4-acyl MPLA (1 sec) 56:0;3O | 1307.8837 | 1.906 | 385.5 | 34.2% | 4.18E+02 | n.d. |
| 4-acyl MPLA (1 sec) 54:0;3O | 1279.8499 | 1.876 | 379.5 | 33.7% | 1.21E+03 | 3.80E+01 |
| 3-acyl DPLA (1 sec) 42:0;2O | 1161.6571 | 1.683 | 341.1 | 36.4% | 2.10E+02 | n.d. |
| 3-acyl DPLA (1 sec) 40:0;2O | 1133.6229 | 1.653 | 335.3 | 21.9% | 9.97E+02 | n.d. |
| 3-acyl MPLA (1 sec) 42:0;2O | 1081.6892 | 1.668 | 338.0 | 100.0% | 4.43E+02 | n.d. |
| 3-acyl MPLA (1 sec) 40:0;2O | 1053.6560 | 1.635 | 332.0 | 100.0% | 1.45E+03 | n.d. |

**Table S5:** Identified lipid A species by MALDI-TIMS-MS/MS in positive ionization mode including accurate mass, mobility, CCS, explained MS2 pattern via custom lipid classes in mzmine, and mobility height for autoclaved and shock-frozen *E. coli* CFT073. Lipid A species annotation was based on accurate mass, fragmentation, and isotopic distribution. CCS values were utilized to confirm phosphorylation sites in MPLA. Species were identified as $[M+Na]^+$ adducts.

| Lipid A species | *m/z* exp | $1/K_0$ [$Vs/cm^2$] | $CCS^{TIMS}$ [$Å^2$] | Explained MS2 intensity | height autoclaved [-] | height shock-frozen [-] |
|---|---|---|---|---|---|---|
| 7-acyl DPLA (3 sec) 98:0;4O | 2058.4283 | 2.498 | 504.2 | 100.0% | n.d. | 6.70E+01 |
| 6-acyl DPLA (2 sec) 84:0;4O | 1848.2315 | 2.340 | 472.1 | 52.0% | n.d. | 2.29E+02 |
| 6-acyl DPLA (2 sec) 82:0;4O | 1820.1980 | 2.312 | 466.5 | 100.0% | n.d. | 9.43E+02 |
| 6-acyl C4'P MPLA (2 sec) 84:0;4O | 1768.2671 | 2.285 | 461.2 | 59.1% | n.d. | 1.10E+02 |
| 6-acyl C1P MPLA (2 sec) 82:0;4O | 1740.2358 | 2.315 | 467.3 | 77.3% | n.d. | 2.50E+02 |
| 6-acyl C4'P MPLA (2 sec) 82:0;4O | 1740.2331 | 2.258 | 455.2 | 76.9% | n.d. | 4.07E+02 |
| 4-acyl C1P MPLA (1 sec) 56:0;3O | 1331.8804 | 1.987 | 402.0 | 54.5% | 1.10E+02 | n.d. |
| 4-acyl C1P MPLA (1 sec) 54:0;3O | 1303.8442 | 1.959 | 396.7 | 100.0% | 4.73E+02 | 1.10E+02 |
| 3-acyl DPLA (1 sec) 40:0;2O | 1157.6187 | 1.715 | 348.3 | 48.1% | 3.20E+02 | n.d. |
| 3-acyl C1P MPLA (1 sec) 42:0;2O | 1105.6833 | 1.752 | 354.7 | 56.6% | 2.80E+02 | n.d. |
| 3-acyl C1P MPLA (1 sec) 40:0;2O | 1077.6506 | 1.721 | 349.5 | 75.0% | 7.33E+02 | n.d. |

## SI-9: Identified lipid A species by LC-TIMS-MS/MS

**Table S6:** Identified lipid A species by LC-TIMS-MS/MS in negative ionization mode including accurate mass, ion type, retention time (RT), mobility, CCS, and mobility height for autoclaved and shock-frozen *E. coli* CFT073. Lipid A species annotation was based on accurate mass, fragmentation, and isotopic distribution. Retention time dependencies were used for confirmation.

| Lipid A species | *m/z* exp | Ion type | RT [min] | $1/K_0$ [Vs/cm²] | $CCS^{TIMS}$ [Å²] | height autoclaved [-] | height shock-frozen [-] |
|---|---|---|---|---|---|---|---|
| 6-Acyl MPLA (2 sec) PEtN 82:0;4O | 1839.2520 | $[M-H]^-$ | 8.62 | 2.328 | 470.8 | 1.01E+01 | 2.17E+02 |
| 6-Acyl DPLA (2 sec) 84:0;4O | 1824.2412 | $[M-H]^-$ | 8.84 | 2.309 | 466.3 | 1.71E+02 | 7.16E+02 |
| 6-Acyl DPLA (2 sec) 82:0;4O | 1796.2105 | $[M-H]^-$ | 8.15 | 2.287 | 461.4 | 2.73E+02 | 4.04E+03 |
| 6-Acyl DPLA (2 sec) 80:0;4O | 1768.1785 | $[M-H]^-$ | 7.86 | 2.263 | 457.0 | 5.08E+01 | 7.52E+02 |
| 6-Acyl MPLA (2 sec) 84:0;4O | 1744.2740 | $[M-H]^-$ | 8.66 | 2.267 | 457.6 | 6.53E+01 | 4.82E+02 |
| 6-Acyl MPLA (2 sec) 82:0;4O | 1716.2440 | $[M-H]^-$ | 8.79 | 2.247 | 453.9 | 6.07E+02 | 6.73E+02 |
| 6-Acyl MPLA (2 sec) 82:0;4O | 1716.2434 | $[M-H]^-$ | 8.40 | 2.243 | 452.9 | 2.23E+02 | 1.96E+03 |
| 6-Acyl MPLA (2 sec) 80:0;4O | 1688.2133 | $[M-H]^-$ | 8.11 | 2.216 | 0.0 | 4.16E+01 | 1.64E+02 |
| 5-Acyl MPLA (2 sec) 68:0;3O | 1490.0492 | $[M-H]^-$ | 7.12 | 2.034 | 411.4 | 4.18E+01 | 2.87E+02 |
| 4-Acyl MPLA (1 sec) 56:0;3O | 1307.8826 | $[M-H]^-$ | 4.99 | 1.894 | 383.6 | 8.63E+02 | 3.83E+01 |
| 4-Acyl MPLA (1 sec) 55:0;3O | 1293.8669 | $[M-H]^-$ | 4.72 | 1.882 | 380.9 | 8.12E+02 | 1.20E+01 |
| 4-Acyl MPLA (1 sec) 54:0;3O | 1279.8508 | $[M-H]^-$ | 4.37 | 1.868 | 378.4 | 4.33E+03 | 1.38E+02 |
| 4-Acyl MPLA (1 sec) 53:0;3O | 1265.8356 | $[M-H]^-$ | 4.08 | 1.861 | 376.8 | 7.91E+02 | n.d. |
| 3-Acyl MPLA (1 sec) 42:0;2O | 1081.6886 | $[M-H]^-$ | 1.80 | 1.657 | 336.6 | 5.37E+02 | 1.40E+01 |
| 3-Acyl MPLA (1 sec) 41:0;2O | 1067.6735 | $[M-H]^-$ | 1.51 | 1.646 | 334.1 | 5.73E+02 | n.d. |
| 3-Acyl MPLA (1 sec) 40:0;2O | 1053.6581 | $[M-H]^-$ | 1.27 | 1.627 | 330.7 | 3.59E+03 | 1.05E+01 |
| 3-Acyl MPLA (1 sec) 39:0;2O | 1039.6418 | $[M-H]^-$ | 1.09 | 1.618 | 328.2 | 4.50E+02 | n.d. |

**Table S7:** Identified lipid A species by LC-TIMS-MS/MS in positive ionization mode including accurate mass, ion type, retention time (RT), mobility, CCS, and mobility height for autoclaved and shock-frozen *E. coli* CFT073. Lipid A species annotation was based on accurate mass, fragmentation, and isotopic distribution. Retention time dependencies were used for confirmation as well as CCS values were utilized to confirm phosphorylation sites in MPLA.

| Lipid A species | *m/z* exp | Ion type | RT [min] | $1/K_0$ [Vs/cm$^2$] | CCS$^{TIMS}$ [Å$^2$] | height autoclaved [-] | height shock frozen [-] |
|---|---|---|---|---|---|---|---|
| 6-Acyl DPLA (2 sec) 82:0;4O | 1820.2046 | $[M+Na]^+$ | 8.14 | 2.327 | 0.0 | 1.30E+03 | 5.02E+03 |
| 4-Acyl C1P MPLA (1 sec) 54:0;3O | 1303.8486 | $[M+Na]^+$ | 4.38 | 1.960 | 396.8 | 1.03E+04 | 1.87E+02 |
| 3-Acyl C1P MPLA (1 sec) 42:0;2O | 1105.6880 | $[M+Na]^+$ | 1.78 | 1.740 | 0.0 | 1.33E+03 | n.d. |
| 3-Acyl C1P MPLA (1 sec) 40:0;2O | 1077.6558 | $[M+Na]^+$ | 1.25 | 1.708 | 347.5 | 5.70E+03 | 3.13E+01 |
| 6-Acyl DPLA (2 sec) 82:0;4O | 1798.2231 | $[M+H]^+$ | 8.13 | 2.349 | 473.7 | 5.28E+03 | 5.39E+04 |
| 6-Acyl C4'P MPLA (2 sec) 82:0;4O | 1718.2580 | $[M+H]^+$ | 8.36 | 2.263 | 0.0 | 3.40E+02 | 9.76E+03 |
| 6-Acyl C1P MPLA (2 sec) 82:0;4O | 1718.2565 | $[M+H]^+$ | 8.66 | 2.311 | 465.4 | 9.11E+02 | 7.10E+03 |
| 5-Acyl C4'P MPLA (2 sec) 68:0;3O | 1492.0620 | $[M+H]^+$ | 7.13 | 2.084 | 421.6 | 2.80E+03 | 3.27E+03 |
| 4-Acyl C1P MPLA (1 sec) 55:0;3O | 1317.8643 | $[M+H]^+$ | 4.68 | 1.977 | 400.7 | 1.57E+03 | n.d. |
| 4-Acyl C1P MPLA (1 sec) 56:0;3O | 1309.8971 | $[M+H]^+$ | 4.96 | 1.973 | 398.4 | 1.70E+04 | 2.22E+02 |
| 4-Acyl C1P MPLA (1 sec) 55:0;3O | 1295.8840 | $[M+H]^+$ | 4.69 | 1.950 | 396.8 | 9.29E+03 | 9.68E+01 |
| 4-Acyl C1P MPLA (1 sec) 53:0;3O | 1289.8333 | $[M+H]^+$ | 4.07 | 1.954 | 0.0 | 1.10E+03 | n.d. |
| 4-Acyl C1P MPLA (1 sec) 54:0;3O | 1281.8661 | $[M+H]^+$ | 4.41 | 1.943 | 393.3 | 8.54E+04 | 3.28E+03 |
| 4-Acyl C1P MPLA (1 sec) 53:0;3O | 1267.8503 | $[M+H]^+$ | 4.06 | 1.934 | 391.3 | 6.61E+03 | n.d. |
| 4-Acyl C1P MPLA (1 sec) 52:0;3O | 1253.8350 | $[M+H]^+$ | 3.71 | 1.919 | 388.9 | 3.87E+03 | n.d. |
| 3-Acyl C1P MPLA (1 sec) 42:0;2O | 1083.7058 | $[M+H]^+$ | 1.82 | 1.753 | 355.8 | 4.81E+03 | n.d. |
| 3-Acyl C1P MPLA (1 sec) 41:0;2O | 1069.6907 | $[M+H]^+$ | 1.49 | 1.736 | 352.1 | 3.46E+03 | n.d. |
| 3-Acyl C1P MPLA (1 sec) 40:0;2O | 1055.6744 | $[M+H]^+$ | 1.25 | 1.698 | 348.6 | 2.54E+04 | 1.24E+02 |
| 3-Acyl C1P MPLA (1 sec) 38:0;2O | 1027.6444 | $[M+H]^+$ | 0.89 | 1.684 | 341.8 | 1.28E+03 | n.d. |

## SI-10: References


(1) Henderson, J. C.; O'Brien, J. P.; Brodbelt, J. S.; Trent, M. S. Isolation and chemical characterization of lipid A from gram-negative bacteria. *JoVE-J. Vis. Exp.* **2013**, e50623.

(2) Sorensen, M.; Chandler, C. E.; Gardner, F. M.; Ramadan, S.; Khot, P. D.; Leung, L. M.; Farrance, C. E.; Goodlett, D. R.; Ernst, R. K.; Nilsson, E. Rapid microbial identification and colistin resistance detection via MALDI-TOF MS using a novel on-target extraction of membrane lipids. *Sci. Rep.* **2020**, *10*, 21536.

(3) Helmer, P. O.; Nordhorn, I. D.; Korf, A.; Behrens, A.; Buchholz, R.; Zubeil, F.; Karst, U.; Hayen, H. Complementing Matrix-Assisted Laser Desorption Ionization-Mass Spectrometry Imaging with Chromatography Data for Improved Assignment of Isobaric and Isomeric Phospholipids Utilizing Trapped Ion Mobility-Mass Spectrometry. *Anal. Chem.* **2021**, *93*, 2135–2143.

(4) Froning, M.; Helmer, P. O.; Hayen, H. Identification and structural characterization of lipid A from *Escherichia coli*, *Pseudomonas putida* and *Pseudomonas taiwanensis* using liquid chromatography coupled to high-resolution tandem mass spectrometry. *Rapid Commun. Mass Spectrom.* **2020**, *34*, e8897.

(5) Schmid, R.; Heuckeroth, S.; Korf, A.; Smirnov, A.; Myers, O.; Dyrlund, T. S.; Bushuiev, R.; Murray, K. J.; Hoffmann, N.; Lu, M.; Sarvepalli, A.; Zhang, Z.; Fleischauer, M.; Dührkop, K.; Wesner, M.; Hoogstra, S. J.; Rudt, E.; Mokshyna, O.; Brungs, C.; Ponomarov, K.; Mutabdžija, L.; Damiani, T.; Pudney, C. J.; Earll, M.; Helmer, P. O.; Fallon, T. R.; Schulze, T.; Rivas-Ubach, A.; Bilbao, A.; Richter, H.; Nothias, L.-F.; Wang, M.; Orešič, M.; Weng, J.-K.; Böcker, S.; Jeibmann, A.; Hayen, H.; Karst, U.; Dorrestein, P. C.; Petras, D.; Du, X.; Pluskal, T. Integrative analysis of multimodal mass spectrometry data in MZmine 3. *Nat. Biotechnol.* **2023**, *41*, 447–449.

(6) Myers, O. D.; Sumner, S. J.; Li, S.; Barnes, S.; Du, X. One Step Forward for Reducing False Positive and False Negative Compound Identifications from Mass Spectrometry Metabolomics Data: New Algorithms for Constructing Extracted Ion Chromatograms and Detecting Chromatographic Peaks. *Anal. Chem.* **2017**, *89*, 8696–8703.

(7) Yang, H.; Smith, R. D.; Chandler, C. E.; Johnson, J. K.; Jackson, S. N.; Woods, A. S.; Scott, A. J.; Goodlett, D. R.; Ernst, R. K. Lipid A Structural Determination from a Single Colony. *Anal. Chem.* **2022**, *94*, 7460–7465.

(8) Guan, X. L.; Loh, J. Y.-X.; Lizwan, M.; Chan, S. C. M.; Kwan, J. M. C.; Lim, T. P.; Koh, T. H.; Hsu, L.-Y.; Lee, B. T. K. LipidA-IDER to Explore the Global Lipid A Repertoire of Drug-Resistant Gram-Negative Bacteria. *Anal. Chem.* **2023**, *95*, 602–611.

(9) Okahashi, N.; Ueda, M.; Matsuda, F.; Arita, M. Analyses of Lipid A Diversity in Gram-Negative Intestinal Bacteria Using Liquid Chromatography-Quadrupole Time-of-Flight Mass Spectrometry. *Metabolites* **2021**, *11*, 197.

(10) Sándor, V.; Dörnyei, Á.; Makszin, L.; Kilár, F.; Péterfi, Z.; Kocsis, B.; Kilár, A. Characterization of complex, heterogeneous lipid A samples using HPLC-MS/MS technique I. Overall analysis with respect to acylation, phosphorylation and isobaric distribution. *J. Mass Spectrom.* **2016**, *51*, 1043–1063.

(11) Sándor, V.; Kilár, A.; Kilár, F.; Kocsis, B.; Dörnyei, Á. Characterization of complex, heterogeneous lipid A samples using HPLC-MS/MS technique III. Positive-ion mode tandem mass spectrometry to reveal phosphorylation and acylation patterns of lipid A. *J. Mass Spectrom.* **2018**, *53*, 146–161.
